# Machine-learned molecular mechanics force fields from large-scale quantum chemical data†

**DOI:** 10.1039/d4sc00690a

**Published:** 2024-06-26

**Authors:** Kenichiro Takaba, Anika J. Friedman, Chapin E. Cavender, Pavan Kumar Behara, Iván Pulido, Michael M. Henry, Hugo MacDermott-Opeskin, Christopher R. Iacovella, Arnav M. Nagle, Alexander Matthew Payne, Michael R. Shirts, David L. Mobley, John D. Chodera, Yuanqing Wang

**Affiliations:** a Computational and Systems Biology Program, Sloan Kettering Institute, Memorial Sloan Kettering Cancer Center New York NY 10065 USA john.chodera@choderalab.org wangyq@wangyq.net; b Pharmaceuticals Research Center, Advanced Drug Discovery, Asahi Kasei Pharma Corporation Shizuoka 410-2321 Japan takaba.kb@om.asahi-kasei.co.jp; c Center for Neurotherapeutics, Department of Pathology and Laboratory Medicine, University of California Irvine CA 92697 USA; d Skaggs School of Pharmacy and Pharmaceutical Sciences, University of California San Diego 9500 Gilman Drive La Jolla CA 92093 USA; e Department of Chemical and Biological Engineering, University of Colorado Boulder Boulder CO 80309 USA; f Open Molecular Software Foundation Davis CA 95618 USA; g Department of Bioengineering, University of California, Berkeley Berkeley CA 94720 USA; h Department of Pharmaceutical Sciences, University of California Irvine California 92697 USA; i Tri-Institutional PhD Program in Chemical Biology, Memorial Sloan Kettering Cancer Center New York 10065 USA; j Simons Center for Computational Physical Chemistry and Center for Data Science, New York University New York NY 10004 USA

## Abstract

The development of reliable and extensible molecular mechanics (MM) force fields—fast, empirical models characterizing the potential energy surface of molecular systems—is indispensable for biomolecular simulation and computer-aided drug design. Here, we introduce a generalized and extensible machine-learned MM force field, espaloma-0.3, and an end-to-end differentiable framework using graph neural networks to overcome the limitations of traditional rule-based methods. Trained in a single GPU-day to fit a large and diverse quantum chemical dataset of over 1.1 M energy and force calculations, espaloma-0.3 reproduces quantum chemical energetic properties of chemical domains highly relevant to drug discovery, including small molecules, peptides, and nucleic acids. Moreover, this force field maintains the quantum chemical energy-minimized geometries of small molecules and preserves the condensed phase properties of peptides and folded proteins, self-consistently parametrizing proteins and ligands to produce stable simulations leading to highly accurate predictions of binding free energies. This methodology demonstrates significant promise as a path forward for systematically building more accurate force fields that are easily extensible to new chemical domains of interest.

## Abstract 2

A generalized and extensible machine-learned molecular mechanics force field trained on over 1.1 million QC data applicable for drug discovery applications. Figure reproduced from the arXiv:201001196 preprint under the arXiv non-exclusive license.

## Graphical abstract

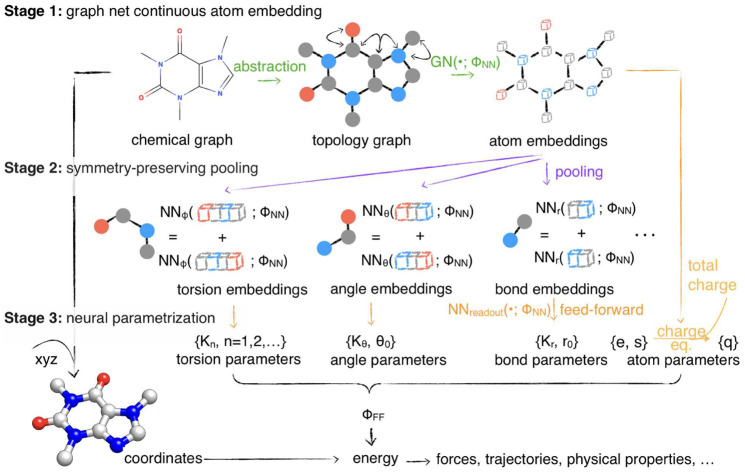

Molecular mechanics (MM) force fields^1,2^ are fast, empirical models that describe the potential energy surfaces of biomolecular systems by treating them as collections of atomic point masses. These point masses interact *via* non-bonded and valence (bond, angle, and torsion) terms, which are typically parametrized to reproduce quantum chemical conformational energetics and physical properties. Despite their simplified representation of the underlying physical model, MM force fields have proven to be indispensable for a multitude of tasks in biomolecular simulation and computer-aided drug design,^3,4^ such as enumeration of putative bioactive conformations,^5^ hit identification *via* virtual screening,^6^ prediction of membrane permeability,^7^ simulations of biomolecular dynamics,^8^ and estimation of protein–ligand binding free energies *via* alchemical free energy calculations.^9^

## 1 Class I MM force fields have been a widely popular compromise between speed and accuracy

Class I MM force fields^1,2^ are most widely used for proteins, lipids, nucleic acids, and other relevant biomolecules due to the computational efficiency afforded by the simple functional form:1
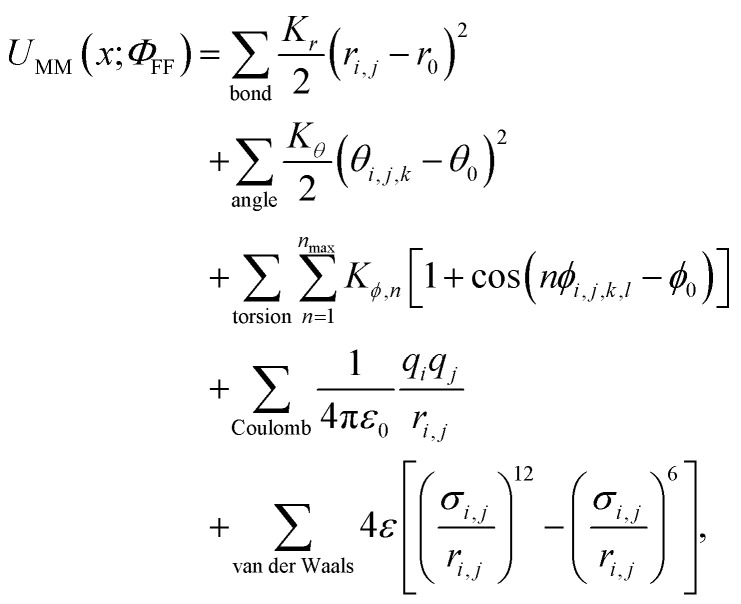
where the total potential energy *U*_MM_ of a molecular system with coordinates *x* is defined by sets of force field parameters *Φ*_FF_ = {*K*_*r*_, *K*_*θ*_, *r*_0_, *θ*_0_, *K*_*ϕ*,*n*_, *ϕ*_0_, *q*, *σ*, *ε*}_*i*_ specified for each atom *i* or valence term (bond, angle, torsion) of the system. An out-of-plane term (an improper torsion) can be also introduced with the torsion term to improve molecular planarity. The van der Waals interactions are usually described with Lennard-Jones 12–6 potentials using the Lorentz–Berthelot^10^ combining rules to determine *σ* and *ε* between different atom types, but alternative combination rules are possible. In practice, such interactions usually require combining distinct force field parameters developed independently for specific chemical domains to complement the heterogeneity of biomolecular systems. Note that the functional forms of force fields can slightly differ among different Class I force fields, incorporating different scaling constants and additional functional terms, such as CMAP^2^ and Urey-Bradley.^1^ The minimalistic nature of Class I force fields has enabled them to achieve extraordinary speed on inexpensive hardware, with modern GPU-accelerated molecular simulation frameworks now able to generate more than 1 microsecond per day for many biomolecular drug targets^11–13^ while still achieving useful accuracy in tasks such as predicting protein–ligand binding free energies for drug discovery.^14–16^

## 2 Traditional MM force field parametrization approaches struggle with complexity, limiting accuracy

Traditionally, the construction of MM force fields requires expert knowledge of physical organic chemistry to build atom-typing rules classifying atoms into discrete categories representing distinct chemical environments, enabling MM parameters to be subsequently assigned from a table of relevant atomic, bond, angle, and torsion parameters. This creates an intractable mixed discrete-continuous optimization problem, posing a labor-intensive challenge, heavily reliant on human effort. Force field accuracy is limited by the resolution of chemical perception, which in turn is limited by the number of distinct atom types. Attempting to improve accuracy by increasing the number of atom types results in a combinatorial explosion of bond, angle, and torsion parameters, which imposes strong practical limits.^17^ As a result, modelers frequently turn to bespoke parameter generation tools—such as Paramfit,^18^ FFBuilder^19^ or OpenFF BespokeFit^20^—to assign individual parameters for molecules of interest for which high accuracy is needed, requiring expensive quantum chemical calculations to be performed *ad hoc* and diminishing the speed benefits of Class I force fields.

## 3 Traditional MM force field parametrization approaches often aim for divide-and-conquer, rather than self-consistency

To tame the explosion of atom type complexity, biomolecular force field efforts have frequently taken the approach of building separate but purportedly compatible models for proteins, small molecules, and other biomolecules independently. For example, the recent AmberTools 23 release^21^ recommends combining independently developed force fields to simulate systems containing proteins,^22^ DNA,^23,24^ RNA,^25^ water,^26–28^ monovalent^29,30^ and divalent^31–33^ counterions, lipids,^34^ carbohydrates,^35^ glycoconjugates,^36,37^ small molecules,^38,39^ post-translational modifications,^40^ and nucleic acid modifications^41^—which collectively might represent more than 100 person-years of effort. While this simplifies the subproblems of parametrizing each class of molecules, using these separate force fields together to treat complex, heterogeneous systems is neither simple nor optimal. There are often overlaps in the chemical space that each force field is designed to model, with no guarantee that the parameters in these regions are identical and remain entirely compatible. This introduces significant caveats when multiple classes of biomolecules interact, risking poor accuracy and greatly frustrating the cases where molecules of different classes must be covalently bonded. As such, extension or expansion to new classes of biomolecules or chemical spaces becomes a time-consuming ordeal, as combining force fields often results in a large combinatorial space of possible force field parameters where the quality of the resulting force field depends heavily on the choices made by the user.

There have been numerous efforts to systematize and automate the process of force field development.^17,19,42–45^ For example, the Open Force Field Initiative has developed a number of modern, open-source tools,^20,46^ datasets, and force fields^44,45^ that employ a direct approach to chemical perception,^17^ which use a standard SMARTS-based chemical substructure query to assign entire sets of valence parameters (atoms, bonds, angles, torsions) in a hierarchical manner, attempting to ameliorate the combinatorial explosion of parameters. There have also been extensive efforts to systematically optimize parameters using finite-difference methods^42,43^ and machine learning approaches.^47,48^ However, much of the work focuses on small molecules, and extending the force field to new chemical domains still requires human effort—jointly optimizing discrete chemical perception rules and continuous force field parameters remains intractable.

## 4 A graph neural network parametrization scheme can automate, simplify, and significantly improve the accuracy of MM force fields with no performance penalty

Recently, we proposed a novel approach—Espaloma^49^ (extensible surrogate potential optimized by mes-sage passing)—which replaces the rule-based discrete atom-typing schemes with a continuous atomic representation generated by graph neural networks that operate on chemical graphs.^49–51^ These atom representations are coupled with a set of symmetry-preserving pooling layers and feed-forward neural networks to enable fully end-to-end differentiable construction of MM force fields. The neural network parameters are optimized directly using standard machine learning frameworks to fit quantum chemical and/or experimental data. The expressiveness of Espaloma's continuous atomic representations eliminates the need to combine force fields developed for different chemical domains (it has been well known^52,53^ that vanilla GNNs cannot realize some crucial local properties such as ring size, whereas in our implementation this is supplemented by chemoinformatics tools) Thus, Espaloma can self-consistently parametrize any system of molecules with elemental coverage in its training set.

Earlier work^49,50^ demonstrated that this approach, in principle, parametrizes multiple classes of biomolecules—the open source Espaloma package was used to train a small Espaloma model for a Class I MM force field on a limited set of 45 000 quantum chemical calculations covering small molecules and amino acids.^49^ While surprisingly robust in comparison to traditional small molecule and amino acid force fields, that model was far from providing comprehensive coverage of chemical space relevant to biomolecular modeling and drug discovery, and its potential usage for real-world applications remained unclear.

## 5 espaloma-0.3: a versatile, robust, and accurate machine-learned Class I MM force field retrainable in a single-GPU day

In this paper, we introduce a significantly enhanced Espaloma framework that incorporates energy and force matching with quantum chemical data, scalability to massive quantum chemical datasets, and stringent regularization for enhanced model stability. We demonstrate how this approach can easily fine-tune the valence terms of an existing Class I small molecule force field (see Section 8 for a discussion on the condensed-phase properties related to the non-bonded parameters) and extend to new chemical domains of interest without a performance penalty, resulting in a generalized and extensible machine-learned Class I MM force field, espaloma-0.3. Trained in a single GPU-day to fit a large and diverse curated quantum chemical dataset of over 1.1 M energy and force calculations for 17 000 unique molecular species, espaloma-0.3 reproduces quantum chemical energetic properties of chemical spaces of small molecules, peptides, and nucleic acids much more accurately than the well-established MM force fields widely used in the fields of biomolecular simulation and computer-aided drug design. Furthermore, it maintains the quantum chemical energy-minimized geometries of small molecules and preserves the condensed phase properties of peptides and folded proteins, thus self-consistently parametrizing proteins and ligands to produce stable simulations leading to highly accurate protein–ligand binding free energy predictions. To our knowledge, this study represents the first well-demonstrated example of a self-consistent MM force field capable of parametrizing a protein–ligand system that is applicable for real-world drug discovery purposes.

This enhanced Espaloma framework demonstrates significant promise as a path forward for systematically building more accurate and extensible force fields with additional quantum chemical data, similarly to how foundational large language models can be fine-tuned to perform better on domain tasks of interest.

## 6 Espaloma provides a flexible, end-to-end differentiable framework for assigning molecular mechanics (MM) parameters using graph neural networks (GNNs)

**Fig. 1:**
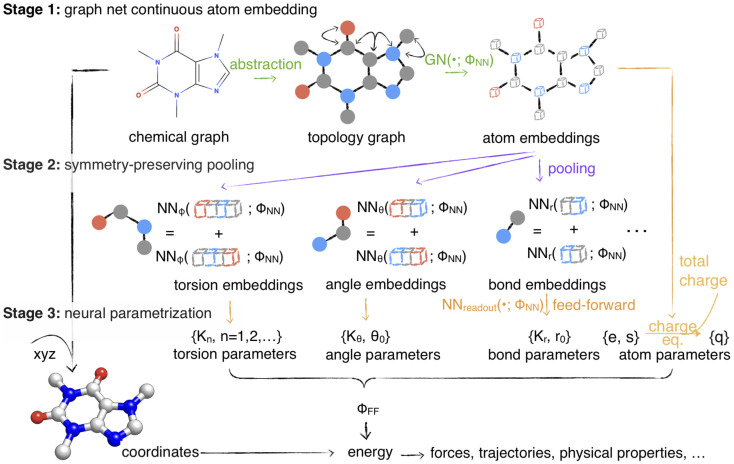
Espaloma is an end-to-end differentiable molecular mechanics parameter assignment scheme for arbitrary organic molecules. Espaloma (extensible surrogate potential optimized by message-passing) is a modular approach for directly computing molecular mechanics force field parameters *Φ*_FF_ from a chemical graph 
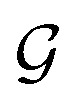
 such as a small molecule or biopolymer *via* a process that is fully differentiable in the model parameters *Φ*_NN_. In Stage 1, a graph neural network is used to generate continuous latent atom embeddings describing local chemical environments from the chemical graph. In Stage 2, these atom embeddings are transformed into feature vectors that preserve appropriate symmetries for atom, bond, angle, and proper/improper torsion inference *via* Janossy pooling.^54^ In Stage 3, molecular mechanics parameters are directly predicted from these feature vectors using feed-forward neural networks. This parameter assignment process is performed once per molecular species, allowing the potential energy to be rapidly computed using standard molecular mechanics or molecular dynamics frameworks thereafter. The collection of parameters *Φ*_NN_ describing the espaloma model can be considered as the equivalent complete specification of a traditional molecular mechanics force field such as GAFF^38,39^/AM1-BCC^55,56^ in that it encodes the equivalent of traditional typing rules, parameter assignment tables, and even partial charge models. Reproduced from ref. 49 with permission from the Royal Society of Chemistry.

Espaloma^49^ (Fig. 1) operates analogously to an atom-typing based force field, where chemical perceptions are predefined to generate MM force field parameters *Φ*_FF_. However, instead of working with atom types, Espaloma operates on a chemical graph 
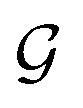
 using a graph neural network (GNN) parametrized by neural network model parameters *Φ*_NN_,2
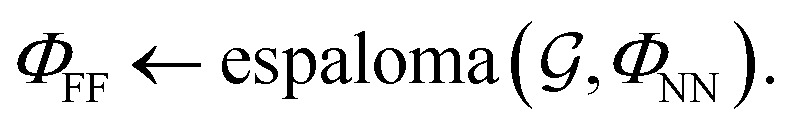


The resulting parameters *Φ*_FF_ can then be subsequently used in a standard molecular mechanics package to compute the MM energy and forces for any conformation, as with a standard MM force field.

Espaloma parametrizes molecular systems in three sequential stages (Fig. 1).

### 6.1 Stage 1

Graph neural networks generate a continuous vectorial atom embedding, replacing discrete atom-typing rules. First, using chemoinformatics toolkits such as RDKit,^57^ the molecular system is abstracted as a graph, with nodes and edges denoted as atoms and covalent bonds, respectively. Espaloma uses GNNs^53,58–66^ as a replacement for rule-based chemical environment perception^17^ to operate on this graph. These neural architectures learn useful representations of atomic chemical environments from simple input features by updating and pooling embedding vectors *via* message-passing iterations to neighboring atoms.^60^ As such, symmetries in chemical graphs (chemical equivalencies) are inherently preserved, while a rich, continuous, and differentiable learnable representation of the atomic environment is derived.

### 6.2 Stage 2

Symmetry-preserving pooling generates continuous bond, angle, and torsion embeddings. The representations determined by GNNs in Stage 1 are used to come up with bond, angle, and torsion representations in a symmetry-preserving manner, where the relevant equivalent atom permutations are listed and summed up *via* Janossy pooling.^54^

### 6.3 Stage 3

Neural parametrization of atoms, bonds, angles, and torsions replaces tabulated parameter assignment. In the final stage, feed-forward neural networks learn the mapping from these symmetry-preserving invariant atom, bond, angle, and torsion embeddings to MM parameters *Φ*_FF_ that reflect the specific chemical environments appropriate for these terms. Each distinct parameter class (such as atom, bond, angle, and torsion parameters) is assigned by a separate neural network, making this stage fully modular. This stage is analogous to the final table lookup step in traditional force field construction, but it offers significant added flexibility due to the continuous embedding that captures the chemical environment specific to the assigned potential energy term.

The final output is a set of force field parameters *Φ*_FF_ uniquely determined by the neural network conditioned on its associated weights *Φ*_NN_. This means that once the *Φ*_NN_ is optimized, biomolecular simulations can be performed as fast as those simulated with traditional MM force fields. Atomic partial charges can also be generated within the Espaloma framework, using a geometry-independent charge equilibration approach^67^ to rapidly generate AM1-BCC^55,56^ quality charges.^68,69^

Overall, the Espaloma framework is end-to-end differentiable—the error in energy (or the function thereof, such as forces) can be backpropagated to optimize the force field parameters *Φ*_FF_, and thereby neural network parameters *Φ*_NN_ that govern how they are produced from the input molecule. Stage 3 is especially modular and flexible. New force field terms that act on atoms, bonds, angles, torsions, or combinations thereof can easily be added and the entire force field refit starting from either an existing *Φ*_NN_ or training from scratch. In this way, Espaloma provides a rapid and flexible approach to experimenting with different potential functions (such as the addition of point polarizability or exploration of alternative functional forms) or retraining with augmented training datasets.

## 7 Extensive open quantum chemical dataset curated to provide coverage of biomolecules: small molecules, proteins, and nucleic acids

To develop a self-consistent MM force field broadly applicable to biomolecular modeling, we first curate a high-quality gas-phase quantum chemical dataset deposited in QCArchive^70^ (Table 1). The curated quantum chemical dataset is built from several components that provide complementary coverage of relevant biomolecular chemistries: from the foundational SPICE dataset,^74^ we extracted a large set of drug-like small molecules selected from PubChem,^78^ dipeptides (capped 2-mers) and their common protonation and tautomeric variants, and diverse molecular fragments providing broad coverage of biomolecules from the DES370K dataset;^76^ from the OpenFF 1.x (“Parsley”)^44^ and 2.x (“Sage”)^45^ datasets, we extracted optimization and torsion-drive datasets for diverse small molecules; a diverse set of dipeptide (capped 2-mers), tripeptides (capped 3-mers), disulfide-bridged, bioactive, and cyclic peptides from the PepConf dataset;^77^ a peptide torsion scan set generated by the Open Force Field Consortium for the OpenFF 3.x (“Rosemary”) force field;^79^ and a new set of RNA nucleosides, trinucleotides, and diverse experimental RNA fragments sourced from the Nucleic Acid Database^80^ and RNA Structure Atlas^81^ to extend coverage to this important and growing class of drug targets.

**Table 1:** Espaloma-0.3 can directly fit quantum chemical potential energies and forces more accurately than baseline force fields**.** Espaloma was fit to quantum chemical (QC) potential energies and forces from various gas-phase QC datasets sourced from QCArchive,^70^ covering a broad chemical space that includes small molecules, peptides, and RNA molecules (see ESI Section B). The entire dataset consists of 17 427 unique molecules and 1 188 317 conformations. These datasets were extracted from three different QCArchive workflows: BasicDataset, OptimizationDataset, and TorsionDriveDataset. The datasets were partitioned into train, validate, and test sets in an 80 : 10 : 10 ratio split by molecules, except for the RNA-Trinucleotide and RNA-Nucleoside datasets. Since RNA nucleosides and trinucleosides lack chemical diversity, the RNA-Nucleoside dataset was used for training, whereas the RNA-Trinucleotide dataset, which covers the same molecules as the RNA-Diverse dataset but with much more diverse conformers, was used as a test set. The number of molecules and total conformations for each dataset is annotated in the table. We report the root mean square error (RMSE) on the training and test sets, along with the performance of other force fields as baselines on the test set. The baseline force fields used were gaff-2.11,^71^ openff-2.0.0,^72^ and openff-2.1.0 (ref. 73) for small molecules, Amber ff14SB^22^ for peptides, and Amber RNA.OL3 (ref. 25) for RNA molecules. All statistics are computed with predicted and reference energies centered to have a zero mean for each molecule similar to the previous work.^49^ The 95% confidence intervals, as annotated in the results, were calculated by bootstrapping molecule replacement using 1000 replicates

Dataset (QCArchive workflow)	Category	Mols	Confs	Split	Espaloma-0.3		Baseline force field (test molecules)				
					Energy RMSE (kcal mol^−1^) force RMSE (kcal mol^−1^ Å^−1^)		Energy RMSE (kcal mol^−1^) force RMSE (kcal mol^−1^ Å^−1^)				
					Train (80%)	Test (10%)	gaff-2.11 (ref. 71)	openff-2.0.0 (ref. 72)		openff-2.1.0 (ref. 73)	ff14SB^22^/RNA.OL3 (ref. 25)
SPICE-Pubchem^74,75^ (dataset)	Small molecule	14 110	608 436	80 : 10 : 10	2.06^2.07^_2.04_	2.30^2.36^_2.25_	4.39^4.48^_4.30_	4.21^4.30^_4.13_	4.45^4.53^_4.37_		—
					6.22^6.26^_6.19_	6.81^6.95^_6.68_	14.02^14.37^_13.71_	13.95^14.20^_13.71_	15.45^15.75^_15.17_		—
SPICE-DES-monomers^74,76^ (dataset)	Small molecule	369	18 435	80 : 10 : 10	1.39^1.46^_1.32_	1.36^1.67^_1.13_	1.88^2.22^_1.57_	2.34^2.75^_1.97_	2.43^2.81^_2.05_		—
					5.86^6.02^_5.69_	5.91^6.42^_5.49_	9.46^10.91^_8.09_	11.12^12.47^_9.86_	11.87^13.15^_10.57_		—
Gen2-Opt (OptimizationDataset)	Small molecule	1024	244 989	80 : 10 : 10	1.36^1.48^_1.26_	1.66^2.29^_1.21_	2.29^2.82^_1.88_	2.18^2.77^_1.73_	2.25^2.85^_1.78_		—
					3.94^4.11^_3.79_	4.47^5.40^_3.90_	10.51^11.36^_9.75_	10.53^11.40^_9.86_	11.67^12.53^_10.83_		—
Gen2-torsion (TorsionDriveDataset)	Small molecule	729	25 832	80 : 10 : 10	1.76^1.91^_1.61_	1.64^2.01^_1.32_	2.53^3.21^_1.95_	1.69^2.06^_1.38_	1.83^2.24^_1.46_		—
					4.31^4.44^_4.18_	4.71^5.29^_4.18_	10.50^11.67^_9.42_	11.11^12.09^_10.21_	11.92^12.87^_11.04_		—
SPICE-dipeptide^74^ (dataset)	Peptide	677	26 279	80 : 10 : 10	3.21^3.26^_3.16_	3.09^3.21^_2.96_	4.24^4.42^_4.07_	4.11^4.28^_3.96_	4.28^4.44^_4.10_		4.36^4.55^_4.20_
					7.98^8.07^_7.88_	7.78^8.02^_7.55_	11.90^12.32^_11.50_	11.95^12.32^_11.62_	11.57^11.88^_11.26_		11.76^12.09^_11.40_
Pepconf-Opt^77^ (OptimizationDataset)	Peptide	557	166 291	80 : 10 : 10	2.61^2.83^_2.43_	2.79^3.13^_2.45_	3.53^3.82^_3.03_	2.91^3.39^_2.56_	3.19^3.66^_2.73_		3.59^4.17^_3.00_
					3.83^4.09^_3.60_	4.01^4.46^_3.63_	8.07^8.23^_7.84_	8.74^9.08^_8.49_	8.79^9.56^_8.27_		9.13^9.70^_8.67_
Protein-torsion (TorsionDriveDataset)	Peptide	62	48 999	80 : 10 : 10	2.27^2.50^_2.06_	1.93^2.14^_1.73_	3.53^3.82^_3.03_	2.91^3.39^_2.56_	3.19^3.66^_2.73_		3.59^4.17^_3.00_
					3.94^4.24^_3.70_	3.49^3.78^_3.22_	8.07^8.23^_7.84_	8.74^9.00^_8.49_	8.79^9.56^_8.27_		9.13^9.70^_8.67_
RNA-diverse (dataset)	RNA	64	3703	80 : 10 : 10	4.12^4.31^_3.95_	4.17^4.52^_3.85_	5.65^6.32^_4.95_	5.79^6.19^_5.37_	6.26^6.90^_5.64_		6.06^6.43^_5.70_
					4.44^4.47^_4.40_	4.41^4.51^_4.29_	17.19^17.71^_16.71_	18.54^19.10^_17.85_	19.68^20.15^_19.19_		19.38^19.83^_18.77_
RNA-trinucleotide (dataset)	RNA	64	35 811	0 : 0:100	—	3.75^3.94^_3.59_	5.79^5.98^_5.61_	5.81^5.96^_5.67_	6.26^6.42^_6.10_		5.94^6.12^_5.77_
					—	4.28^4.39^_4.20_	17.15^17.28^_17.00_	18.88^19.02^_18.72_	19.97^20.13^_19.81_		19.82^19.97^_19.67_
RNA-nucleoside (dataset)	RNA	4	9542	100 : 0:0	1.32^1.49^_1.16_	—	—	—	—		—
					4.17^4.47^_3.86_	—	—	—	—		—

To capture the rugged conformational energy surface of biomolecules, the quantum chemical datasets were extracted from three different QCArchive workflows: Dataset, OptimizationDataset, and TorsionDriveDataset. A Dataset contains single-point energy calculations of structures that are not necessarily at their local quantum energy minima, generated using MD simulations or conformer generators. An OptimizationDataset is a collection of QM optimization trajectories for a given structure. A TorsionDriveDataset involves torsion scans performed on a set of rotatable torsions, followed by QM optimization.

The curated dataset consists of 1 188 317 conformations of 17 427 unique molecules in total. We also computed the AM1-BCC ELF10 partial charges using the OpenEye Toolkits to train and generate AM1-BCC^55,56^ quality partial charges with Espaloma. Complete details of the dataset construction and composition are given in ESI Section B.† All quantum chemical energies are computed with the Open Force Field (OpenFF) standard level of quantum chemical theory (B3LYP-D3BJ/DZVP),^44,45^ which balances the computational efficiency and accuracy to reproduce the conformations generated by higher levels of theories.^82^ These quantum chemical datasets were generated with the open source psi4 quantum chemistry packag^83^ using the QCArchive^70^ QCFractal infrastructure *via* OpenFF QCSubmit^84^ workflows.

## 8 Espaloma force field reproduces quantum chemical energies and forces

Leveraging the curated gas-phase quantum chemical datasets discussed in Section 2, we fine-tune and extend the OpenFF 2.0 (“Sage”) force field, openff-2.0.0—a Class I MM force field originally developed for small molecules—into new chemical domains of interest, resulting in a novel Class I MM force field termed espaloma-0.3. Similar to the original implementation^49^ and historic practice in MM force field parametrization,^22,24,25,43,44,85^ we optimized the valence parameters (bonds, angles, and proper/improper torsions) and use the Lennard-Jones parameters from openff-2.0.0.^45^ While it is possible to optimize Lennard-Jones parameters as well, it is critical to include more computationally expensive condensed-phase simulations when doing so.^86,87^ For partial charges, following the protocol of Wang *et al.*^68^ we predict the electronegativity and hardness of atoms used in a charge equilibration^67^ to predict atomic partial charges while preserving the total charge of a given molecule. We utilize the AM1-BCC ELF10 partial charges computed with the OpenEye Toolkits as our target partial charges.

We enhance the original Espaloma framework to improve the model stability and data efficiency (see ESI Section C† for further details):

• quantum chemical forces are incorporated into training to provide more information about the quantum chemical potential surface;

• L2 regularization is applied to proper and improper torsion force constants to suppress spurious features in torsion profiles;

• improper torsion terms expressed using *n* = 1, 2 periodicities to reduce the complexity of the model and to align with other conventional force fields which usually employs *n* = 1, 2 periodicities;

• node features that were sensitive to resonance form have been eliminated to ensure chemically equivalent representations of the same molecule receive identical parameters.

To train espaloma-0.3, we randomly shuffle the datasets and split each dataset by molecules into train, test, and validation sets (80%, 10%, and 10%, respectively) based on unique isomeric SMILES strings. Since the MM force field is incapable of reproducing quantum chemical heats of formation, which are reflected as an additive offset in quantum chemical energy targets for each molecule, we shift the reference quantum chemical energy of each molecule to have zero mean; note that when deployed, the absolute value of MM energy is not physically meaningful and traditional MM force fields are never used to simulate bond-breaking events. The loss function used in training included deviations from quantum chemical snapshot energies and forces, as well as deviations from target partial charges for each molecule in the training set (see ESI Section C† for complete details).

As shown in Table 1, espaloma-0.3 significantly outperforms all baseline force fields (gaff-2.11,^71^ openff-2.0.0,^72^ openff-2.1.0,^73^ Amber ff14SB,^22^ Amber RNA. OL3 (ref. 25)) in reproducing quantum chemical energies and forces, demonstrating the ability of espaloma-0.3 to recapitulate the quantum chemical energy surface more accurately than current-generation Class I MM potentials for biomolecules and organic chemistry despite using the same functional form. In contrast, the baseline force fields widely popular in the field of biomolecular simulations yield considerable energy errors and huge force errors (on average twice to thrice that of espaloma-0.3) with respect to quantum chemical calculations. The performance superiority holds true across diverse chemical categories, suggesting the general utility of espaloma-0.3 in a wide array of chemical and biochemical modeling tasks, as evidenced in Sections 11 and 12. These observations hold true when Espaloma is trained with different data splitting strategies (ESI Table 1†).

Notably, the backbone torsion parameters for ff14SB are empirically adjusted to improve agreement with condensed-phase NMR data. Therefore, it might be expected to perform less effectively when benchmarked against quantum chemical energetic properties. For a more rigorous comparison, we conducted the same benchmark experiment using ff14SBonlysc,^88^ which is the same model as ff14SB but without the empirical backbone corrections. The resulting energy RMSE on test datasets for SPICE-Dipeptide, Pepconf-Opt, and Protein-Torsion were 4.36 [95% CI: 4.52, 4.19], 3.93 [95% CI: 3.58, 4.23], and 3.59 [95% CI: 3.00, 4.18] kcal mol^−1^ respectively, with corresponding force RMSE values of 11.76 [95% CI: 11.41, 12.12], 10.22 [95% CI: 9.82, 10.68], 9.13 [95% CI: 8.67, 9.70] kcal mol^−1^ Å^−1^; espaloma-0.3 performed superiorly better for all three datasets.

## 9 Espaloma force field preserves quantum chemical energy minima

We next examined whether the ability of espaloma-0.3 to quantitatively reproduce the quantum chemical equilibrium conformational energetics extends to an ability to qualitatively preserve the conformations of quantum chemical local energy minima—important for accurately representing geometries for phenomena like ligand binding docking studies, simulations, or free energy calculations. To assess this, we used a standardized industry benchmark of gas-phase QM-optimized geometries (the OpenFF Industry Benchmark Season 1 v1.1 (ref. 89)‡‡https://github.com/openforcefield/qca-dataset-submission/tree/master/submissions/2021-06-04-OpenFF-Industry-Benchmark-Season-1-v1.1. obtained from QCArchive) to compare the structures and energetics of conformers optimized using espaloma-0.3 and baseline force fields (openff-2.0.0, openff-2.1.0, and gaff-2.11) with respect to their QM-optimized geometries at the B3LYP-D3BJ/DZVP level of theory. The dataset is a collection of drug-like molecules selected by industry partners of the Open Force Field Consortium and is representative of their current interests in chemical spaces, serving as an out-of-distribution test dataset. It contains 9728 unique molecules and 73 301 conformers after filtering out any quantum chemical calculation failures due to convergence issues and connectivity changes during geometry optimization.

**Fig. 2:**
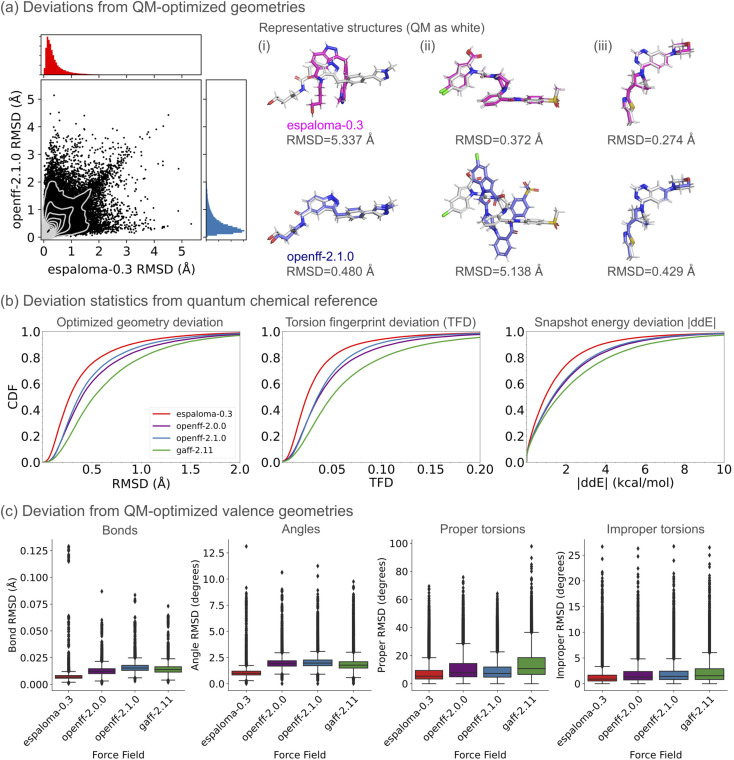
Espaloma-0.3 preserves the location of quantum chemical energy minima. An industry standard benchmark of gas-phase QM-optimized geometries (the OpenFF Industry Benchmark Season 1 v1.1 (ref. 89) from QCArchive), comprising 9728 unique molecules and 73 301 conformers, was used to compare the structures and energetics of conformers optimized using espaloma-0.3, openff-2.0.0,^72^ openff-2.1.0,^73^ and gaff-2.11 (ref. 71) with respect to their QM-optimized geometries at the B3LYP-D3BJ/DZVP level of theory. (a) Representative scatter plot of root-mean-square deviation (RMSD) of atomic positions between espaloma-0.3 and openff-2.1.0. The superposed structures between the QM-optimized (white) and MM-optimized geometries with the maximum RMSD obtained by (i) espaloma-0.3, (ii) openff-2.1.0, and (iii) the median RMSD of espaloma-0.3 are shown. (b) The cumulative distribution functions of root-mean-square deviation (RMSD) of atomic positions, torsion fingerprint deviation (TFD) score, and relative energy differences (ddE) as described in a previous work^90^ are reported. (c) Distributions of bond, angle, proper torsion, and improper torsion RMSD within each conformer with respect to its QM-optimized geometries are shown as quartile box plots. Lower values for all metrics indicate that the MM-optimized geometry is close to the quantum chemical reference structure.

As shown in Fig. 2(a and b), the geometries and relative conformer energies with respect to their quantum chemical reference values showed better agreement with espaloma-0.3 than with the baseline force fields—openff-2.0.0, openff-2.1.0, and gaff-2.11. Additionally, the bonds, angles, and torsions in MM-optimized geometries obtained using espaloma-0.3 show close agreement with quantum chemical values (Fig. 2(c)), resulting in an overall performance compatible or slightly better than the baseline force fields. The bond outliers (>0.1 Å) with espaloma-0.3 arise from three sulfonamides connected to aliphatic carbons, comprising a total of 30 conformers—0.04% of the conformers in the entire benchmark dataset—exhibiting ∼0.4 Å elongated S–N bond distances in the sulfonamide groups compared to the QM-optimized geometries (ESI Fig. 4(a)†). 12 other molecules containing sulfonamide groups, excluding the bond RMSD outliers were found within the benchmark dataset with each molecular conformer featuring reasonable bond distances within the sulfonamide group (ESI Fig. 4(b)†). However, the nitrogen geometry of pyrazoles and imidazoles substituted with sulfonamides became trigonal pyramidal when minimized with espaloma-0.3, rather than preserving a flat ring geometry and losing their sp2 hybridized features, as observed with QM-optimized geometries (ESI Fig. 4(c)†). The angle outlier is also related to a sulfonamide but was a singleton of a non-druglike molecule containing a single conformer, with ∼40° deviation from its original QM-optimized geometry (ESI Fig. 4(a)†).

Nonetheless, the degree of improvement of espaloma-0.3 relative to openff-2.0.0 is surprising and intriguing, considering that the Lennard-Jones parameters are transferred from openff-2.0.0 and the overlap in the underlying Optimization and TorsionDrive datasets used for optimizing both force fields. This is notable, despite espaloma-0.3 was trained on quantum chemical dataset comprising larger and broader chemical species.

## 10 Espaloma force field reproduces experimental NMR observables for peptides and folded proteins

### 10.1 Peptides

To quantitatively assess the ability of espaloma-0.3 to model the intrinsic backbone preferences of amino acids, we performed MD simulations of thirteen short, unstructured peptides for which NMR observables have been experimentally measured.^91,92^ The peptides are composed of 3 to 5 residues, uncapped, and have protonated C Termini due to the low pH of the NMR experiments. Measured vicinal scalar couplings inform on the backbone dihedral preferences of these peptides. Scalar couplings were computed from 500 ns trajectories using a Karplus model,^98,102,104–107^ and agreement with experimental observables was quantified using a *χ*^2^ value.

**Fig. 3:**
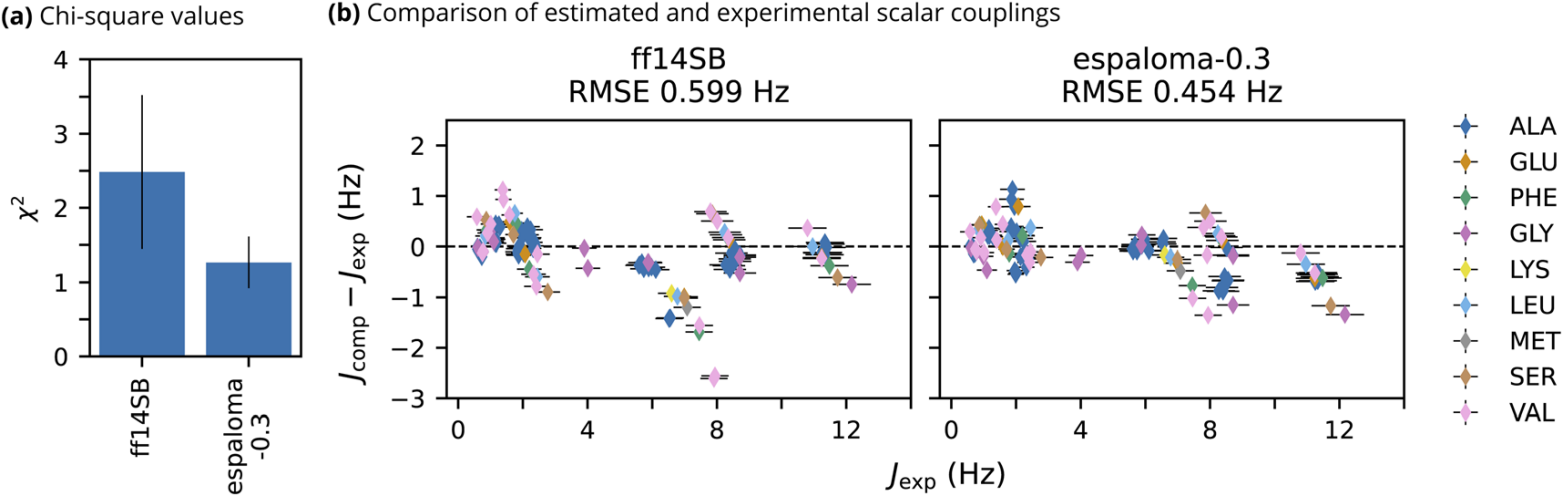
espaloma-0.3 reproduces experimental NMR scalar couplings of unstructured peptides better than well-established biomolecular force field, ff14sb. (a) *χ*^2^ values (lower is better) quantifying deviations of simulated NMR scalar couplings computed from 500 ns trajectories from experimental NMR measurements.^91,92^ Error bars represent a 95% confidence interval constructed from the critical values of a Student's *t* distribution and the standard error of the mean across the NMR observables. (b) Comparison of the error in computed estimates of NMR scalar couplings *versus* experiment. Colors represent the identity of the amino acid associated with each scalar coupling. Horizontal error bars represent the estimate of the systematic error in the experimental scalar coupling, and vertical error bars represent the uncertainty due to the computed estimate (standard error of the mean across 3 replicates) and the uncertainty due to the experimental value (systematic error) added in quadrature.

Overall, espaloma-0.3 produces closer agreement with experiment than ff14SB, as evidenced by the low *χ*^2^ value (Fig. 3(a)). With note, ff14SB tends to exhibit closer agreement with experiments on amino acids with short side chains such as glycine and alanine (Fig. 3(b)). This is unsurprising as the backbone torsion parameters for ff14SB were tuned to reproduce the NMR scalar couplings for the alanine 5-mer peptide included in this dataset.^22^ However, espaloma-0.3 tends to have closer agreement with experiments on more challenging amino acids with charged (*e.g.* lysine), bulky (*e.g.* methionine), or β-branched (*e.g.* valine) side chains.

### 10.2 Folded proteins

**Fig. 4:**
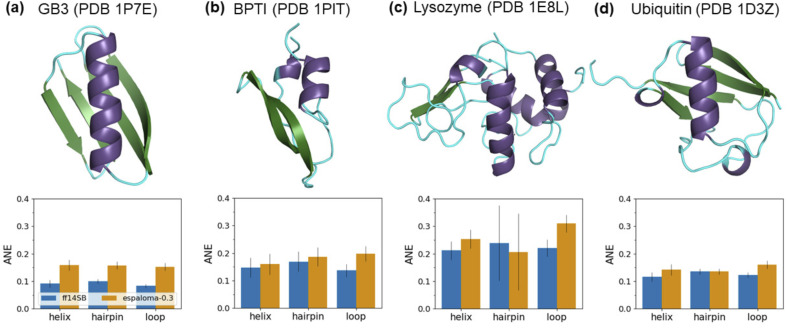
Espaloma-0.3 reproduces experimental NMR scalar couplings of folded globular proteins with a slightly higher error compared to the well-established biomolecular force field, ff14sb. The absolute normalized error (ANE) values (lower the better),^22^ quantifying the deviations of simulated NMR scalar couplings from 10 μs trajectories compared to experimental measurements,^93–103^ are compared for (a) GB3, (b) BPTI, (c) lysozyme, and (d) ubiquitin. The regions of helix, hairpin, and loop are depicted in purple, green, and cyan, respectively, as defined by Ramachandran angles from the crystal structures. Error bars represent a 95% confidence interval, constructed from the critical values of a Student's *t*-distribution and the standard error of the mean across the NMR observables, based on three replicates of the 10 μs simulation. Note that ANEs were applied instead of *χ*^2^ values to address the potential underestimation of experimental and Karplus model inaccuracies, as well as the significant variance in the scalar coupling value range across different coupling types,^22^ resulting in a more intuitive metric (see ESI Section E.2† for more details). A comparison with *χ*^2^ values can be found in ESI Table 2.†

The intrinsic dynamics of both the backbone and *χ*_1_ side chains in folded proteins were assessed by conducting MD simulations of four globular proteins: the third IgG-binding domain of protein G (GB3), bovine pancreatic trypsin inhibitor (BPTI), lysozyme, and ubiquitin (Fig. 4). These proteins, for which scalar couplings have been measured by NMR experiments, have been extensively studied for the development of protein force fields.^22^ Scalar couplings were computed from 10 μs trajectories using the same Karplus model^96,98,102,104–109^ that was employed in the peptide analysis. Additionally, inter-residue scalar couplings between backbone–backbone hydrogen bonds^109^ were computed for GB3 and ubiquitin. The agreement with experimental observables was quantified using an average normalized error (ANE) metric, motivated by the work of Maier *et al.*^22^ The ANE metric was introduced to address the potential underestimation of experimental errors and Karplus model inaccuracies, as well as the significant variance in the scalar coupling value range across different coupling types, resulting in a more intuitive metric than the *χ*^2^ value (see ESI Section E.2† for more details). Here, 0 indicates the best possible agreement, while 1 indicates maximum deviation.

**Fig. 5:**
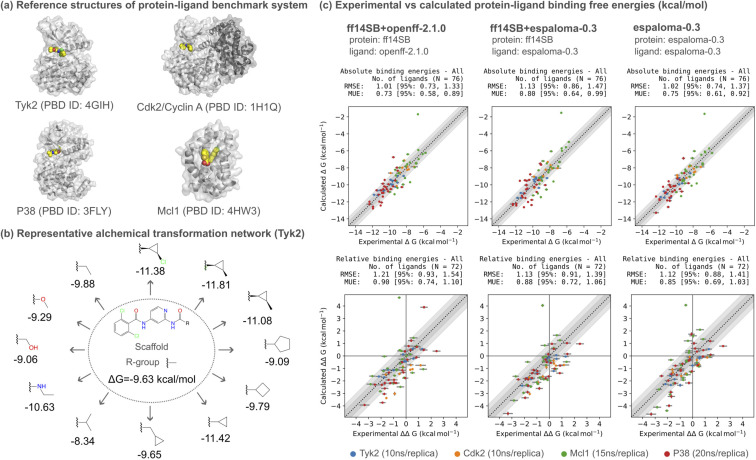
espaloma-0.3 can be used for accurate protein–ligand alchemical free energy calculations. (a) Protein–ligand (PL) alchemical free energy calculations were calculated for Tyk2 (10 ns/replica), Cdk2 (10 ns/replica), Mcl1 (15 ns/replica), P38 (20 ns/replica) using a curated PL-benchmark dataset (see ESI Section F†) which comprises 76 ligands in total. The PL structures used to setup the alchemical free energy calculations for each target system is shown. Here, we used Perses 0.10.1 relative free energy calculation infrastructure,^110^ based on OpenMM 8.0.0,^111^ to assess the accuracy of espaloma-0.3 and openff-2.1.0 (ref. 73) combined with Amber ff14SB force field^22^ for comparison. (b) Schematic illustration of the alchemical ligand transformation network for Tyk2. The methyl R-group in the center is alchemically transformed into various R-groups. The binding free energy for each R-group is annotated alongside the respective R-groups. (c) The openff-2.1.0 (ref. 73) with protein parametrized with Amber ff14SB force field (ff14SB + openff-2.1.0) achieves an absolute free energy (Δ*G*) RMSE of 1.01 [95% CI: 0.73, 1.33] kcal mol^−1^. The espaloma-0.3 for predicting valence parameters and partial charges of small molecules combined with Amber ff14SB force field for proteins (ff14SB + espaloma-0.3) achieves an absolute free energy (Δ*G*) RMSE of 1.13 [95% CI: 0.86, 1.47] kcal mol^−1^. Parametrizing small molecule and protein self-consistently with espaloma-0.3 (espaloma-0.3) achieves absolute free energy (Δ*G*) RMSE of 1.02 [95% CI: 0.74, 1.37] kcal mol^−1^ which is comparable to those obtained by (ff14SB + openff-2.1.0) and (ff14SB + espaloma-0.3). All systems were solvated with TIP3P water^26^ and neutralized with 300 mM NaCl salt using Joung and Cheatham monovalent counterions.^29^ The light and dark gray regions depict the confidence bounds of 0.5 kcal mol^−1^ and 1.0 kcal mol^−1^, respectively.

Overall, espaloma-0.3 accurately replicates experimental NMR scalar couplings for all four folded proteins, but with slightly higher ANE values compared to ff14sb (ESI Table 2, Fig. 5† and 4). The larger deviations tend to arise from the side chain scalar couplings (ESI Fig. 6†). Simulations with espaloma-0.3 also indicate a greater decrease in the occupancy of defined folded regions, such as the alpha (α) and beta (β) backbone structures (ESI Fig. 7†), leading to slightly increased backbone flexibility, as suggested by the Cα RMSD plots (ESI Fig. 8†).

Although folded proteins tend to be more flexible and less reproducible regarding the experimental NMR scalar couplings when simulated with espaloma-0.3 than with ff14sb, the above results, along with the peptide benchmark results, reflect the transferability of Espaloma's neural network parameters—which were trained on gas-phase quantum chemistry data—to the condensed phase.

## 11 Espaloma force field accurately describes protein–ligand binding free energies

To evaluate espaloma-0.3 for real-world drug discovery applications, we performed relative alchemical free energy calculations on a curated protein–ligand binding benchmark dataset, which was adopted from the Open Force Field protein–ligand benchmark dataset (see ESI Section F†).§§https://github.com/openforcefield/protein-ligand-benchmark/tree/d3387602bbeb0167abf00dfb81753d8936775dd2. We selected target systems from available datasets based on several criteria: firstly, we prioritized systems with ligands that can be effectively modeled to alleviate the potential sampling issues arising from poor initial ligand poses; secondly, we excluded systems with cofactors and ions near the ligand binding site to simplify the evaluation; thirdly, we considered systems with diverse structure–activity relationships, including ligand net charges, multiple R-group enumeration, and scaffold hopping. As a result, we selected four well-studied protein–ligand binding benchmark systems. The protein structures, ligand poses, and ligand transformation networks were manually curated to ensure the free energy benchmark was an accurate and reproducible assessment of force field accuracy.

• Tyk2 (PDB: 4GIH),^112^ a non-receptor tyrosine-protine kinase, has therapeutic significance in inflammatory bowel diseases (IBD). This particularly popular system has good convergence and served as a control experiment.

• Cdk2 (PDB: 1H1Q),^113^ a cyclin-dependent kinase, is involved in molecular pathology of cancer and is, therefore, a popular target for structure-based drug design. We use this system, which is complexed with cyclin A, to test the capability of parametrizing multiple protein subunits.

• P38 (PDB: 3FLY)^114^ is a mitogen-activated protein (MAP) kinase which is a central component in signaling networks in mammalian cell types. This target is another well-studied system, but is expected to be more challenging compared to Tyk2 and Cdk2 because of the larger ligand transformations and exploration of structure–activity relationships with multiple R-groups from different scaffold positions.

• Mcl1 (PDB: 4HW3)^115^ (myeloid cell leukemia 1) is a member of the Bcl-2 family of proteins, which is overexpressed in various cancers and promotes aberrant survival of tumor cells. This target entails all ligands with a net charge of −1 and includes scaffold hopping; thus, chosen to test the capability to simulate free energy calculations for charged ligands and scaffold hopping.

Within each system, we benchmarked three approaches of parametrization to evaluate the accuracy of espaloma-0.3 in modeling either the ligand alone or the entire protein–ligand complex:

• Protein: ff14SB/ligand: openff-2.1.0 (ff14SB + openff-2.1.0): as a baseline, we parametrize the ligand region using a well-established small molecule force field openff-2.1.0 (ref. 73) and use the Amber ff14SB^22^ to parametrize the protein.

• Protein: ff14SB/ligand: espaloma-0.3 (ff14SB + espaloma-0.3): we parametrize the ligand region using espaloma-0.3 and use the Amber ff14SB^22^ to parametrize the protein. We only parametrize the ligand region with espaloma-0.3 to provide a head-to-head comparison with openff-2.1.0.

• Protein: espaloma-0.3/ligand: espaloma-0.3 (espaloma-0.3): we apply espaloma-0.3 to both the ligand and protein regions of the system. This is to test the capability of espaloma-0.3 to entirely replace the force field parametrization pipeline. Instead of using two separate force fields for small molecules and proteins, each developed independently, we aim to apply a self-consistently developed force field that covers different chemical domains.

As our training dataset does not yet include water and ions, all systems were solvated with TIP3P water^26^ and neutralized with the Joung and Cheatham monovalent counterions.^29^ The perses 0.10.1 infrastructure^110^ was used to perform the alchemical protein–ligand binding free energy calculations (see ESI Section G†).

In Fig. 5 and Table 2, we illustrate that espaloma-0.3, which parametrizes both the protein and ligand self-consistently, has comparable protein–ligand binding free energy performance to ff14SB + openff-2.1.0. espaloma-0.3 achieves absolute (Δ*G*) and relative (ΔΔ*G*) free energy RMSE of 1.02 [95% CI: 0.74, 1.37] kcal mol^−1^ and 1.12 [95% CI: 0.88, 1.41] kcal mol^−1^, respectively. Correspondingly, the Δ*G* and ΔΔ*G* RMSE for ff14SB + openff-2.1.0 were 1.01 [95% CI: 0.73, 1.33] kcal mol^−1^ and 1.21 [95% CI: 0.93, 1.54] kcal mol^−1^, respectively. Although, the reported error and correlation statistics have overlapping confidence intervals, these results are encouraging as espaloma-0.3 demonstrates its capability to cover different chemical domains, which traditional force fields have struggled for decades and have not accomplished.

Protein–ligand alchemical free energy calculation benchmarks show espaloma-0.3 achieves high accuracy that is competitive to well-established force fields**.** Here, we report several different metrics to assess the performance of the protein–ligand binding benchmark results including root mean square error (RMSE), mean unsigned error (MUE), the square of the correlation coefficient (*R*^2^), and the Spearman's rank correlation coefficient (*ρ*) along with 95% CI for each metric. The initial PDB ID, number of compounds, number of edges (ligand transformations), the binding affinity range, and the simulation time per replica are reported in the table

**Table:** 

System	PDB ID	Compds	Edges	Range (kcal mol^−1^)	ns/replica	Protein: ff14SB/ligand: openff-2.1.0					
						Relative (ΔΔ*G*)		Absolute (Δ*G*)			
						RMSE	MUE	RMSE	MUE	*R* ^2^	Spearman *ρ*
Tyk2	4GIH	13	12	3.47	10	0.54^0.71^_0.36_	0.45^0.62^_0.28_	0.50^0.64^_0.36_	0.42^0.57^_0.27_	0.80^0.93^_0.53_	0.89^0.96^_0.75_
Cdk2	1H1Q	10	9	2.78	10	1.43^1.75^_1.04_	1.29^1.67^_0.80_	0.74^0.93^_0.50_	0.63^0.86^_0.41_	0.48^0.85^_0.13_	0.69^0.92^_0.30_
Mcl1	4HW3	25	24	4.19	15	1.50^2.12^_0.83_	1.02^1.55^_0.63_	1.36^2.01^_0.77_	0.97^1.41^_0.66_	0.50^0.73^_0.35_	0.71^0.86^_0.57_
P38	3FLY	28	27	3.81	20	1.06^1.30^_0.81_	0.87^1.09^_0.65_	0.90^1.19^_0.60_	0.69^0.92^_0.50_	0.57^0.78^_0.38_	0.76^0.89^_0.63_

**Table:** 

System	PDB ID	Compds	Edges	Range (kcal mol^−1^)	ns/replica	Protein: ff14SB/ligand: espaloma-0.3					
						Relative (ΔΔ*G*)		Absolute (Δ*G*)			
						RMSE	MUE	RMSE	MUE	*R* ^2^	Spearman *ρ*
Tyk2	4GIH	13	12	3.47	10	0.70^0.98^_0.34_	0.52^0.80^_0.28_	0.48^0.65^_0.29_	0.37^0.55^_0.23_	0.79^0.95^_0.49_	0.89^0.97^_0.71_
Cdk2	1H1Q	10	9	2.78	10	1.15^1.44^_0.85_	1.05^1.36^_0.73_	0.56^0.74^_0.32_	0.46^0.66^_0.27_	0.63^0.92^_0.27_	0.80^0.96^_0.53_
Mcl1	4HW3	25	24	4.19	15	1.38^1.96^_0.90_	1.06^1.44^_0.76_	1.51^2.15^_0.90_	1.08^1.56^_0.74_	0.60^0.80^_0.42_	0.77^0.90^_0.63_
P38	3FLY	28	27	3.81	20	1.03^1.26^_0.81_	0.82^1.05^_0.59_	1.10^1.32^_0.86_	0.88^1.13^_0.63_	0.38^0.64^_0.11_	0.62^0.80^_0.34_

**Table:** 

System	PDB ID	Compds	Edges	Range (kcal mol^−1^)	ns/replica	Protein: espaloma-0.3/ligand: espaloma-0.3					
						Relative (ΔΔ*G*)		Absolute (Δ*G*)			
						RMSE	MUE	RMSE	MUE	*R* ^2^	Spearman *ρ*
Tyk2	4GIH	13	12	3.47	10	0.67^0.87^_0.45_	0.56^0.76^_0.35_	0.46^0.58^_0.33_	0.40^0.53^_0.28_	0.81^0.94^_0.64_	0.90^0.97^_0.79_
Cdk2	1H1Q	10	9	2.78	10	0.84^1.05^_0.58_	0.75^0.99^_0.51_	0.63^0.76^_0.48_	0.58^0.74^_0.41_	0.47^0.82^_0.14_	0.68^0.90^_0.41_
Mcl1	4HW3	25	24	4.19	15	1.44^1.99^_0.96_	1.10^1.50^_0.76_	1.40^2.09^_0.78_	1.00^1.43^_0.67_	0.56^0.78^_0.40_	0.75^0.88^_0.63_
P38	3FLY	28	27	3.81	20	1.02^1.24^_0.77_	0.79^1.04^_0.56_	0.91^1.13^_0.68_	0.75^0.95^_0.57_	0.47^0.68^_0.24_	0.68^0.82^_0.49_

Notably, a large outlier for the Mcl1 system for all three cases was observed as shown in Fig. 5. The problematic ligand transformation and the initial ligand pose is illustrated in ESI Fig. 11.† The relative binding affinity ΔΔ*G* computed with ff14SB + espaloma-0.3 was 4.05 kcal mol^−1^ (Fig. 5(b)). However, we found that the error can be reduced to 2.60 kcal mol^−1^ when the alchemical binding free energy calculation was performed from a flipped binding pose, which is in better agreement with the experimental difference (ΔΔ*G* = −0.54 kcal mol^−1^).

We also conducted another set of free energy calculations for the four target systems, each with three parametrization approaches (ESI Fig. 9†). In most cases, the absolute (Δ*G*) and relative (ΔΔ*G*) binding free energies from the two independent trials were within 1.0 kcal mol^−1^, demonstrating reasonable reproducibility; except for P38, which tends to be a more challenging target for the free energy calculations to reproduce.

It is worth noting that the ligands from the protein–ligand binding benchmark dataset are highly dissimilar to the molecules used in developing espaloma-0.3, with a maximum Tanimoto similarity of 0.5 between the two sources, suggesting the high generalizability of Espaloma (ESI Fig. 10†).

### 11.1 Regularization and larger training dataset significantly improve performance

To assess the impact of dataset scale and the regularization procedures introduced here for training espaloma-0.3, we compared the protein–ligand binding free energy calculations using the first-generation Espaloma force field (0.2.2),^49^ which was trained on a limited quantum chemical dataset and without regularization compared to 0.3. The free energy calculations were conducted for all four target systems and were prepared similarly to those described above. In ESI Fig. 12,† espaloma-0.2.2 significantly underperforms compared to espaloma-0.3 for the Cdk2 system due to a large outlier. espaloma-0.2.2 also demonstrates lesser performance on the Tyk2 system, as illustrated in ESI Fig. 13.† Importantly, the protein–ligand binding free energy calculations were unstable for Mcl1 and P38, with many of the ligand transformations being suspended during the simulation. These results indicate that espaloma-0.3, trained on an extensive quantum chemical dataset and with an improved training strategy, has resulted in the development of a robust and stable Espaloma force field.

## 12 Espaloma force field produces stable long-time molecular dynamics of protein–ligand complex system

Recent benchmarks of machine learned force fields demonstrated that many of these potentials are accurate but cannot produce stable molecular dynamics simulations.^116^ To assess whether espaloma-0.3 was sufficiently stable and robust for general use in molecular dynamics simulations, we performed multiple replicates of a 3 microsecond MD simulation of a solvated protein–ligand complex (Tyk2 complexed with ligand #1, ESI Fig. 13†) and monitored the root-mean square deviation (RMSD) of the ligand and Cα protein atoms, as well as the root-mean square fluctuation (RMSF) profiles of the Cα protein atoms, as shown in ESI Fig. 14.†

The simulations parametrized with espaloma-0.3 remained comparably stable to those generated with ff14SB + openff-2.1.0, with both protein and ligand RMSD generally remaining below 2.0 Å. The averaged RMSF profiles, simulated using espaloma-0.3 and ff14SB + openff-2.1.0, showed a similar trend, with a Pearson correlation coefficient of 0.76. However, espaloma-0.3 exhibited higher peaks, indicating greater protein flexibility with this force field—a finding that aligns with those described in Section 5.

## 13 Discussion

In this study, we introduced an enhanced graph neural network approach to rapidly construct a new generation of accurate, robust, and generalizable machine-learned MM force field, espaloma-0.3, capable of fine-tuning and extending to new chemical domains of interest. The newly developed force field captures both quantitative and qualitative behavior of quantum chemical conformational energetics for a wide range of chemical species. As a result, it not only recapitulates quantum chemical conformational energetics and geometries, but it also reproduces experimental NMR observables for peptides and folded proteins, leading to accurate predictions of protein–ligand binding free energies when both the protein and ligand are self-consistently parametrized with espaloma-0.3. We hope this work will lay the foundations to inspire the design of new generations of machine learning-empowered molecular mechanics force fields that can self-consistently describe the wide chemical domains relevant to biomolecular modeling and drug discovery.

### 13.1 An open chemically and conformationally diverse quantum chemical dataset was curated to construct espaloma-0.3

In this paper, we have curated a high-quality open dataset covering chemical spaces and conformational regions of interest to biomolecular modeling, including small molecules, peptides, and RNA. We demonstrated how our enhanced Espaloma framework can scale to foundational quantum chemical datasets, enabling the achievement of a stable machine-learned MM force field. We released this dataset along with our implementation in the hope that this will enable the community to further optimize MM force fields by building on this dataset, or fine-tuning the espaloma-0.3 model with additional data much the way foundational large language models (LLMs) can be fine-tuned to perform better on domain tasks of interest.

### 13.2 Espaloma-0.3 quantitatively and qualitatively recapitulates quantum chemical conformational energy landscapes

We demonstrated that current force fields typically exhibit considerable disagreement with quantum chemical calculations in terms of reproducing conformational energies and forces (Table 1). With carefully crafted training and regularization strategies, we show that espaloma-0.3 not only quantitatively agrees more closely with quantum chemical conformational energetics for a wide variety of chemical species, but also behaves qualitatively similarly with quantum chemistry, even in low data regimes (ESI Fig. 1†). Although espaloma-0.3 poses a challenge in preserving the quantum chemical energy minima for some sulfonamide groups (ESI Fig. 4†), more rigorous hyperparameter tuning of the Espaloma framework may help resolve this problem, especially adjusting the weights for each loss component, as we find this to be sensitive to the overall performance.

### 13.3 Chemical diversity and high-energy conformers are important for accurately capturing quantum chemical energies and forces with Espaloma

The cross-validation experiment (ESI Fig. 2†), in which Espaloma is trained without certain categories of chemical species (small molecules, peptides, or RNA), suggests that quantum chemical datasets with broad chemical coverage—specifically, the SPICE-Pubchem (small molecules) dataset—can perceive and extrapolate the chemical environments for out-of-distributed chemical domains. A lack of chemical diversity leads to large quantum chemical force errors, whereas reproducing energies is easier (ESI Fig. 2(a)†). Similarly, cross-validating certain dataset classes (single-point energies generated by MD [Dataset], optimization trajectories of enumerated conformers [OptimizationDataset], or one-dimensional torsion drives [TorsionDriveDataset]) suggests that high-energy conformers may be important to accurately capture the quantum chemical energies and forces with Espaloma and other machine learning-based methods (ESI Fig. 2(b)†).^117^ The quantum chemical forces of peptide datasets, including local energy minima conformers (Pepconf-Opt dataset from [OptimizationDataset]), were poorly reproduced when trained without datasets storing relatively high energy conformers (SPICE-Dipeptide dataset from [Dataset]).

### 13.4 Espaloma-0.3 can be easily extended to other chemical spaces of interest

The chemical space covered by an Espaloma force field can easily be extended to spaces highly relevant in other areas of biomolecular modeling, such as lipids, DNA, and glycans, by simply augmenting the quantum chemical dataset used in training. In constructing espaloma-0.3, we demonstrated that this approach easily scales to 1.1 million energies and forces, representing nearly 17 000 chemical species, in less than a single GPU-day. Because loss function is easily parallelizable, this approach should scale gracefully to much larger datasets by simply distributing gradient computation across multiple GPUs, enabling rapid parametrization on much larger datasets or extension to new chemical domains of interest.

### 13.5 Espaloma offers a modular and extensible approach to building MM force fields

Since the Espaloma architecture and loss function are modular^49^ and, as demonstrated here, new force fields can be trained in a single GPU-day, Espaloma offers the opportunity to rapidly explore different MM functional forms. For example, many molecular mechanics simulation packages support atom-pair specific 1–4 Lennard-Jones and electrostatic parameters, alternative Lennard-Jones mixing rules, or alternative functional forms for van der Waals treatment. Of particular interest are Class II force fields,^1,2^ where higher-order couplings between valence terms are introduced to reproduce the bond and angle vibrations more accurately—while the combinatorial explosion of these terms presents a problem for atom type based force fields, Espaloma does not suffer from the same issue and may provide a robust way to parametrize these force fields.

### 13.6 Espaloma fit to condensed-phase properties can further improve accuracy

While we have demonstrated the ability to create a force field capable of reproducing NMR observables for peptides and folded proteins, as well as predicting accurate protein–ligand binding free energies solely from fitting to quantum chemical data, further assessment is needed to confirm its ability to accurately reproduce condensed-phase properties. Since non-bonded interactions are generally optimized to fit condensed-phase properties, training against these properties may be necessary to further improve the predictive accuracy of such properties. An earlier study has shown that optimizing against condensed-phase mixture properties, rather than properties of pure systems, is better suited to improve force field accuracy for biomolecular systems.^87^ The end-to-end differentiable nature of Espaloma makes it possible to employ reweighting approaches to directly fit to experimental free energies or thermodynamics^118–120^ or other thermophysical properties.^87^ This could either be done directly during fitting or during a second-stage fine-tuning procedure that adapts an Espaloma force field to specific applications of interest by jointly fitting the valence and non-bonded terms. The challenge of this endeavor lies in the difficulty of analytically taking derivatives of condensed phase properties w.r.t. the force field, and thereby the neural network parameters, in order to constrain them within the physically feasible range.

### 13.7 Quantifying force field uncertainty could help generate more robust force fields

One of the challenges in force field development is quantifying the contribution of errors in the force field to predicted quantities. While statistical error is generally reported, this systematic force field error is frequently the major source of error in biomolecular simulations. In recent years, several approaches have emerged to quantify uncertainty in deep learning methods, including mean-variance estimation, Bayesian methods, and ensemble methods.^121,122^ Employing these methods to propagate force field uncertainty into predicted free energies and physical properties could enable Espaloma to provide a quantitative assessment of force field uncertainty. With a better understanding of how this uncertainty propagates to task predictions, we envision that uncertainty-based active learning^123^ or adversarial attacks^124^ could be employed to identify the most valuable new data to be generated in future efforts to train more robust Espaloma force fields.¶¶Implementation, experiment, and dataset details, as well as additional results, are deferred to the ESI, where ref. 125–151 are cited.

## Code availability

The Python code to download the quantum chemical data from QCArchive is available from https://github.com/choderalab/download-qca-datasets. The scripts used to train and evaluate espaloma-0.3 is available from https://github.com/choderalab/refit-espaloma. The scripts used to perform the small molecule geometry benchmark is available from https://github.com/choderalab/geometry-benchmark-espaloma. The curated protein–ligand benchmark dataset can be found from https://github.com/kntkb/protein-ligand-benchmark-custom, and the scripts to perform and analyze the alchemical protein–ligand binding affinity calculation with Perses is available from https://github.com/choderalab/pl-benchmark-espaloma-experiment. The scripts used to perform the MD simulation of Tyk2 protein–ligand system is available from https://github.com/choderalab/vanilla-espaloma-experiment. These python codes are also summarized in https://github.com/choderalab/espaloma-0.3.0-manuscript. The code used for the peptide benchmark study is available from https://github.com/openforcefield/proteinbenchmark.

## Data availability

The raw quantum chemical datasets downloaded from QCArchive is deposited in Zenodo (https://zenodo.org/record/8148817). The pre-processed input data used to train espaloma-0.3 is deposited in Zenodo (https://zenodo.org/record/8150601). The QM- and MM-minimized structures used for the small molecule geometry benchmark study is deposited in Zenodo (https://doi.org/10.5281/zenodo.8378216).

## Author contributions

Conceptualization: KT, YW, JDC; methodology: KT, YW; investigation of espaloma RMSE metric: KT, YW; investigation of small molecule geometry benchmark: KT, PKB; investigation of peptide benchmark: CEC; investigation of globular protein benchmark: AJF, CEC, KT; investigation of protein–ligand binding free energy calculation: KT; investigation of protein–ligand standard MD simulation: KT; software: KT, YW, IP, MMH, HM, CRI; writing – original draft: KT, YW; writing – review & editing: KT, IP, PKB, CEC, AJF, MMH, HM, CRI, AMN, AMP, MRS, DLM, JDC, YW; funding acquisition: JDC; resources: JDC; supervision: JDC, YW.

## Conflicts of interest

J. D. C. is a current member of the Scientific Advisory Board of OpenEye Scientific Software, Redesign Science, Ventus Therapeutics, and Interline Therapeutics, and has equity interests in Redesign Science and Interline Therapeutics. The Chodera laboratory receives or has received funding from multiple sources, including the National Institutes of Health, the National Science Foundation, the Parker Institute for Cancer Immunotherapy, Relay Therapeutics, Entasis Therapeutics, Silicon Therapeutics, EMD Serono (Merck KGaA), AstraZeneca, Vir Biotechnology, Bayer, XtalPi, Interline Therapeutics, the Molecular Sciences Software Institute, the Starr Cancer Consortium, the Open Force Field Consortium, Cycle for Survival, a Louis V. Gerstner Young Investigator Award, and the Sloan Kettering Institute. A complete funding history for the Chodera lab can be found at http://choderalab.org/funding. Y. W. has limited financial interests in Flagship Pioneering, Inc. and its subsidiaries. M. R. S. is an Open Science Fellow with Psivant Sciences and consults for Relay Therapeutics. D. L. M. serves on the scientific advisory boards of Anagenex and OpenEye Scientific Software, Cadence Molecular Sciences, and is an Open Science Fellow with Psivant.

## Supplementary Material

SC-015-D4SC00690A-s001
