# Supplementary material for: Machine-learned molecular mechanics force fields from large-scale quantum chemical data
Source: Chem Sci. 2024 Jun 26;15(32):12861–78. doi: 10.1039/d4sc00690a (PMC11322960; doi:10.1039/d4sc00690a)
Supplement: SC-015-D4SC00690A-s001 [file SC-015-D4SC00690A-s001.pdf]

Electronic Supplementary Material (ESI) for Chemical Science.
This journal is © The Royal Society of Chemistry 2024

Appendix: Machine-learned molecular mechanics force fields for the simulation of protein-ligand systems and beyond.

Kenichiro Takaba (ORCID: 0000-0002-2481-8830)[1, 2], Anika J. Friedman (ORCID: 0000-0002-5427-2779)[3], Chapin E. Cavender (ORCID: 0000-0002-5899-7953)[4], Pavan Kumar Behara (ORCID: 0000-0001-6583-2148)[4], Iván Pulido (ORCID: 0000-0002-7178-8136)[1], Michael M. Henry (ORCID: 0000-0002-3870-9993)[1], Hugo MacDermott-Opeskin (ORCID:0000-0002-7393-7457)[6], Christopher R. Iacovella (ORCID:0000-0003-0557-0427)[1], Arnav M. Nagle (ORCID: 0009-0002-6749-4917)[7, 1], Alexander Matthew Payne (ORCID: 0000-0003-0947-0191)[1, 9], Michael R. Shirts (ORCID: 0000-0003-3249-1097)[3], David L. Mobley (ORCID: 0000-0002-1083-5533)[8], John D. Chodera (ORCID: 0000-0003-0542-119X)[1], Yuanqing Wang (ORCID: 0000-0003-4403-2015)[10, 1]

[1]Computational and Systems Biology Program, Sloan Kettering Institute, Memorial Sloan Kettering Cancer Center, New York, N.Y. 10065, United States; [2]Pharmaceuticals Research Center, Advanced Drug Discovery, Asahi Kasei Pharma Corporation, Shizuoka 410-2321, Japan; [3]Center for Neurotherapeutics, Department of Pathology and Laboratory Medicine, University of California, Irvine, CA 92697, United States; [4]Skaggs School of Pharmacy and Pharmaceutical Sciences, University of California San Diego, 9500 Gilman Drive, La Jolla, CA, 92093, United States; [5]Department of Chemical and Biological Engineering, University of Colorado Boulder, Boulder, CO, 80309, United States; [6]Open Molecular Software Foundation, Davis CA 95618, United States; [7]Department of Bioengineering, University of California, Berkeley, Berkeley, CA, 94720, United States; [8]Department of Pharmaceutical Sciences, University of California, Irvine, California 92697, United States; [9]Tri-Institutional Ph.D. Program in Chemical Biology, Memorial Sloan Kettering Cancer Center, New York, New York 10065, United States; [10]Simons Center for Computational Physical Chemistry and Center for Data Science, New York University, New York, N.Y. 10004, United States

***For correspondence:**
takaba.kb@om.asahi-kasei.co.jp (KT); john.chodera@choderalab.org (JDC); wangyq@wangyq.net (YW)

A Code dependencies

Core dependencies include a modified version of Espaloma 0.3.0 release [49] (https://github.com/choderalab/espaloma/tree/4c6155b72d00ce0190b3cb551e7e59f0adc33a56), PyTorch 1.1.2 [125], Deep Graph Library 0.9.0 [64], and Open Force Field Toolkit 0.10.6 [126], to refit and evaluate the espaloma model. A modified version of Openmmforcefields 0.11.0 [127] (https://github.com/kntkb/openmmforcefields/tree/6d2c3dcd33d9800a32032d28b6b2dca92f348a43) was used to run all the relative alchemical protein-ligand binding free energy calculations with Perses 0.10.1 infrastructure [110]. Espaloma 0.2.4 release and a modified version of Espaloma 0.3.0 was used to parametrize small molecules with `espaloma-0.2.2` and `espaloma-0.3`, respectively. A modified version of Perses 0.10.1 (https://github.com/kntkb/perses/tree/0d069fc1cf31b8cce1ae7a1482c3fa46bc1382d2) was used to self-consistently parametrize both small molecules and proteins with `espaloma-0.3`. A modified version of cinnabar 0.3.0 [128] (https://github.com/kntkb/cinnabar/tree/de7bc6623fb25d75848aa1c9f538b77cd02a4b01) was used to support arbitrary tick frequency when plotting ΔG and $\Delta\Delta G$ plots.

B MolSII QCArchive quantum chemical datasets

The Python code used to download the quantum chemical (QC) datasets from the MolSSI QCArchive [80] is available at https://github.com/choderalab/download-qca-datasets. The QC datasets utilized in this study were obtained from various workflows implemented in the QCArchive ecosystem, including `Dataset`, `OptimizationDataset`, and `TorsionDriveDataset` generated at the B3LYP-D3BJ/DZVP level of theory.

This level of theory was chosen to maintain consistency with the Open Force Field Consortium [44, 45], and it is expected to balance computational efficiency and accuracy in reproducing conformations generated by higher-level theories [78].

The QC datasets in **Table 1** are composed of the following datasets deposited in QCArchive and annotated based on their respective categories.

Small molecules

- **SPICE-Pubchem** [71] [3] [4] [5] [6] [7] [8] is a collection of `Dataset` that contains a comprehensive and diverse collection of small, drug-like molecules obtained from Pubchem [84]. It includes atoms within the range of 3 to 50, including hydrogens, and encompasses the elements of Br, C, Cl, F, H, I, N, O, P, and S.
- **SPICE-DES-Monomers** [71] [9] is a `Dataset`, sourced from DES370K [73], consists of small molecules (up to 22 atoms) chosen to cover a wide range of chemical space, including the elements of Br, C, Cl, F, H, I, N, O, P, and S.
- **Gen2-Opt** [10] [11] [12] [13] [14] is a collection of `OptimizationDataset` that contains drug-like molecules used for the parametrization of the OpenFF 1.2.0 ("Parsley") [44] small molecule force field developed by the Open Force Field Consortium. This dataset is one of the datasets used to generate the first generation espaloma force field, `espaloma-0.2.2`.

[3]Source: https://github.com/openforcefield/qca-dataset-submission/tree/master/submissions/2021-11-08-QMDataset-pubchem-set1-single-points
[4]Source: https://github.com/openforcefield/qca-dataset-submission/tree/master/submissions/2021-11-08-QMDataset-pubchem-set2-single-points
[5]Source: https://github.com/openforcefield/qca-dataset-submission/tree/master/submissions/2021-11-09-QMDataset-pubchem-set3-single-points
[6]Source: https://github.com/openforcefield/qca-dataset-submission/tree/master/submissions/2021-11-09-QMDataset-pubchem-set4-single-points
[7]Source: https://github.com/openforcefield/qca-dataset-submission/tree/master/submissions/2021-11-09-QMDataset-pubchem-set5-single-points
[8]Source: https://github.com/openforcefield/qca-dataset-submission/tree/master/submissions/2021-11-09-QMDataset-pubchem-set6-single-points
[9]Source: https://github.com/openforcefield/qca-dataset-submission/tree/master/submissions/2021-11-15-QMDataset-DES-monomers-single-points
[10]Source: https://github.com/openforcefield/qca-dataset-submission/tree/master/submissions/2020-03-20-OpenFF-Gen-2-Optimization-Set-1-Roche
[11]Source: https://github.com/openforcefield/qca-dataset-submission/tree/master/submissions/2020-03-20-OpenFF-Gen-2-Optimization-Set-2-Coverage
[12]Source: https://github.com/openforcefield/qca-dataset-submission/tree/master/submissions/2020-03-20-OpenFF-Gen-2-Optimization-Set-3-Pfizer-Discrepancy
[13]Source: https://github.com/openforcefield/qca-dataset-submission/tree/master/submissions/2020-03-20-OpenFF-Gen-2-Optimization-Set-4-eMolecules-Discrepancy
[14]Source: https://github.com/openforcefield/qca-dataset-submission/tree/master/submissions/2020-03-20-OpenFF-Gen-2-Optimization-Set-5-Bayer

- **Gen2-Torsion** [15] [16] [17] [18] [19] [20] [21] [22] [23] [24] [25] [26] is a collection `TorsionDriveDataset` that contains torsion scans of drug-like molecules which is part of the dataset used for the parametrization of the OpenFF 2.0.0 ("Sage") [45] small molecule force field developed by the Open Force Field Consortium.

Peptides

- **SPICE-Dipeptide** [71] [27] is a `Dataset` that contains a broad coverage of the possible dipeptides capped with ACE and NME groups formed by the 20 natural amino acids and their common protonation variants. This includes two forms of CYS (neutral or negatively charged), two forms of GLU (neutral or negatively charged), two forms of ASP (neutral or negatively charged), two forms of LYS (neutral or positively charged), and three forms of HIS (neutral forms with a hydrogen on either ND1 or NE2, and a positively charged form with hydrogens on both).
- **Pepconf-Opt** [28] is a `OptimizationDataset` that contains short peptides, including capped, cyclic, and disulfide-bonded peptides originally sourced from Prasad et al. [74] and regenerated by the Open Force Field Consortium. In this study, the `default-dlc` QC specification was utilized, differing from the one used in the first generation espaloma force field (`espaloma-0.2.2`) [49], leading to improved chemical convergence.
- **Protein-torsion** [29] [30] [31] [32] is a collection of `TorsionDriveDataset` that contains various torsion scans of polypeptides (capped 1-mers and capped 3-mers) generated by the Open Force Field Consortium for the OpenFF 3.x ("Rosemary") force field [75]. These torsion scans cover χ_1 and χ_2 angles in the rotatable side chains, as well as ϕ, ψ, and ω angles in the backbones.

RNA

- **RNA-Diverse** [33] is a `Dataset` that contains comprehensive and diverse collection of experimental RNA structures. It includes 138 base pair structures and 295 base triple structures sourced from the Nucleic Acid Database [76]. Additionally, the dataset contains 4056 representative trinucleotide structures

[15] Source: https://github.com/openforcefield/qca-dataset-submission/tree/master/submissions/2020-03-12-OpenFF-Gen-2-Torsion-Set-1-Roche

[16] Source: https://github.com/openforcefield/qca-dataset-submission/tree/master/submissions/2020-03-23-OpenFF-Gen-2-Torsion-Set-1-Roche-2

[17] Source: https://github.com/openforcefield/qca-dataset-submission/tree/master/submissions/2020-03-12-OpenFF-Gen-2-Torsion-Set-2-Coverage

[18] Source: https://github.com/openforcefield/qca-dataset-submission/tree/master/submissions/2020-03-23-OpenFF-Gen-2-Torsion-Set-2-Coverage-2

[19] Source: https://github.com/openforcefield/qca-dataset-submission/tree/master/submissions/2020-03-12-OpenFF-Gen-2-Torsion-Set-3-Pfizer-Discrepancy

[20] Source: https://github.com/openforcefield/qca-dataset-submission/tree/master/submissions/2020-03-23-OpenFF-Gen-2-Torsion-Set-3-Pfizer-Discrepancy-2

[21] Source: https://github.com/openforcefield/qca-dataset-submission/tree/master/submissions/2020-03-12-OpenFF-Gen-2-Torsion-Set-4-eMolecules-Discrepancy

[22] Source: https://github.com/openforcefield/qca-dataset-submission/tree/master/submissions/2020-03-23-OpenFF-Gen-2-Torsion-Set-4-eMolecules-Discrepancy-2

[23] Source: https://github.com/openforcefield/qca-dataset-submission/tree/master/submissions/2020-03-12-OpenFF-Gen-2-Torsion-Set-5-Bayer

[24] Source: https://github.com/openforcefield/qca-dataset-submission/tree/master/submissions/2020-03-26-OpenFF-Gen-2-Torsion-Set-5-Bayer-2

[25] Source: https://github.com/openforcefield/qca-dataset-submission/tree/master/submissions/2020-03-12-OpenFF-Gen-2-Torsion-Set-6-supplemental

[26] Source: https://github.com/openforcefield/qca-dataset-submission/tree/master/submissions/2020-03-26-OpenFF-Gen-2-Torsion-Set-6-supplemental-2

[27] Source: https://github.com/openforcefield/qca-dataset-submission/tree/master/submissions/2021-11-08-QMDataset-Dipeptide-single-points

[28] Source: https://github.com/openforcefield/qca-dataset-submission/tree/master/submissions/2020-10-26-PEPCONF-Optimization

[29] Source: https://github.com/openforcefield/qca-dataset-submission/tree/master/submissions/2021-11-18-OpenFF-Protein-Dipeptide-2D-TorsionDrive

[30] Source: https://github.com/openforcefield/qca-dataset-submission/tree/master/submissions/2022-02-10-OpenFF-Protein-Capped-1-mer-Sidechains

[31] Source: https://github.com/openforcefield/qca-dataset-submission/tree/master/submissions/2022-05-30-OpenFF-Protein-Capped-3-mer-Backbones

[32] Source: https://github.com/openforcefield/qca-dataset-submission/tree/master/submissions/2023-02-06-OpenFF-Protein-Capped-3-mer-Omega

[33] Source: https://github.com/openforcefield/qca-dataset-submission/tree/master/submissions/2022-07-07-RNA-basepair-triplebase-single-points

obtained from the RNA Structure Atlas website [77], where the experimentally observed internal and hairpin loop motifs, as well as junction loops of representative sets of RNA 3D Structures with an X-ray resolution cutoff of 2.5 Å, were segmented into all possible trinucleotide permutations, resulting in 64 unique molecules. These trinucleotide structures are capped with O5' hydroxyl groups at the 5' end and clustered to select the representative structures. For the espaloma refitting experiment, only the trinucleotides were utilized.

- **RNA-Trinucleotide** [34] is a `Dataset` that provides a broader and more diverse structural coverage of trinucleotides compared to the **RNA-Diverse** dataset.
- **RNA-Nucleoside** [35] is a `Dataset` that comprises a comprehensive and diverse collection of nucleosides (adenosine, guanosine, cytidine, and uridine) without O5' hydroxyl atoms. These nucleosides are generated using 500 K implicit solvent MD and torsion scanning on N-glycosidic bond (χ torsion) that connects the base and sugar, resulting in diverse sugar pucker conformations and extensive coverage of χ torsions.

C Espaloma refitting experiment

The Python code used to refit and evaluate `espaloma-0.3` is available at https://github.com/choderalab/refit-espaloma. It should be noted that `espaloma-0.3` is no longer compatible with `espaloma-0.2.x` models and vice versa.

C.1 Data preparation

The quantum chemical datasets obtained from the QCArchive [70] in **SI Section B** were preprocessed prior to the refitting experiment. Molecules with a gap between the minimum and maximum energy larger than 0.1 Hartree (62.5 kcal/mol) were excluded. Since the van der Waals parameters affect the physical property prediction, which is computationally challenging to optimize, we focus on optimizing the valence parameters and use `openff-2.0.0` force field [45] for the van der Waals parameters. AM1-BCC [55, 56] ELF10 [36] partial charges were pre-computed using the OpenEye Toolkits as reference charges. These charges were then used to predict the atomic partial charges based on the predicted electronegativity and hardness of atoms, following the same protocol described in the earlier works by Wang et al. [49]. To ensure that each molecule was represented only once, duplicate molecules across different datasets were merged, ensuring that unique molecules were distributed among the train, validate, or test dataset.

C.2 Machine learning experimental details

C.2.1 Input features

One of the improvements made from the previous Espaloma framework [49] is the exclusion of resonance-sensitive features, such as valences and formal charges, in order to improve the handling of molecules with atomic resonance, such as guanidinium and carboxylic acid. In this study, the input features of the atoms included the one-hot encoded element, as well as the hybridization, aromaticity, ring membership of sizes 3 to 8, atom mass, and the degree of the atoms, which is defined as the number of directly-bonded neighbors, all assigned using the RDKit 2023-03-4 release package [130].

C.2.2 Data splitting and augmentation

To handle molecular graphs with varying numbers of conformers, all molecules were divided into sets of 50 conformers during training. If there were fewer than 50 conformers, additional ones were randomly selected to reach a total of 50 conformers. This enabled mini-batching with randomized molecules, making

[34] Source: https://github.com/openforcefield/qca-dataset-submission/tree/master/submissions/2022-10-21-RNA-trinucleotide-single-points

[35] Source: https://github.com/openforcefield/qca-dataset-submission/tree/master/submissions/2023-03-09-RNA-nucleoside-single-points

[36] ELF10 denotes that the ELF ("electrostatically least-interacting functional groups") conformer selection process was used to generate 10 diverse conformations from the lowest energy 2% of conformers. Electrostatic energies are assessed by computing the sum of all Coulomb interactions in vacuum using the absolute values of MMFF charges assigned to each atom [129]. AM1-BCC charges [55, 56] are generated for each conformer and then averaged.

the training process more stochastic compared to the previous study [49], where the mini-batch was applied to set of molecules with the same number of conformers rather than individual molecules.

C.2.3 Hyperparameter optimization

The hyperparameters were briefly optimized utilizing a subset of data from **SI Section B**, which included `OpenFF Gen2-Opt`, `SPICE-Dipeptide`, and `RNA-Diverse` datasets. The data was partitioned into train : validate : test sets in a 40:30:30 ratio. During the training process, energy and force matching were applied, along with partial charge fitting using the charge equilibrium approach [49, 69].

$$\mathcal{L} = W_{\texttt{energy}}\mathcal{L}_{\texttt{energy}} + W_{\texttt{force}}\mathcal{L}_{\texttt{force}} + W_{\texttt{charge}}\mathcal{L}_{\texttt{charge}} \tag{3}$$

Following the protocol specified in Wang et al. [49], we utilized GraphSAGE [131] as the graph neural network model, the Adam optimizer [132], and the Rectified Linear Unit (ReLU) activation function, while maintaining the energy and charge loss weights to 1 and 1e-3, respectively, throughout the optimization experiment. The hyperparameters subject to optimization included the batch size (32, 64, 128, 256), the depth of the graph neural network (2, 3, 4, 5), the depth of the Janossy pooling network (2, 3, 4, 5), the learning rates (1e-3, 1e-4, 5e-5, 1e-5), the number of units per layer (64, 128, 256, 512), and the force weights (1, 1e-1, 1e-2, 1e-3, 1e-4) via grid search on the validation set, and trained for 3000 epochs for each optimization experiment.

As a result, the optimal configuration was determined as follows: For the atom embedding stage (**Stage1**), three GraphSAGE layers with 512 units and ReLU activation function were employed. For the symmetry preserving pooling stage (**Stage2**) and the readout stage (**Stage3**), we used four feed-forward layers with 512 units and ReLU activation, a learning rate of 1e-4, and a force loss weight of 1.

C.2.4 Production run

The datasets from **SI Section B** were partitioned into train, validate, and test sets with a distribution of 80:10:10 ratio, respectively, with few exceptions. Notably, the entire `RNA-Nucleoside` dataset was exclusively utilized for the train set, while the entire `RNA-Trinucleoside` dataset was allocated for the test set. This partitioning scheme was designed to incorporate diverse molecular structures and enable a comprehensive evaluation of the performance of the espaloma model.

It should be noted that the espaloma model (`espaloma-0.3-rc1`), trained with the hyperparameters described above, reproduced torsion profiles poorly compared to its quantum chemical reference structures (**SI Figure 3**). We found that this problem could be remedied by truncating the improper torsion terms to only $n = 1, 2$ periodicities, instead of $n = 1, ..., 6$ as in the original method [49], and by utilizing regularization for the proper and improper torsion force constants. Regarding these findings, the final espaloma model was trained with the following loss function with all weights set to 1:

$$\mathcal{L} = W_{\texttt{energy}} * \mathcal{L}_{\texttt{energy}} + W_{\texttt{force}} * \mathcal{L}_{\texttt{force}} + W_{\texttt{proper}} * \mathcal{L}_{\texttt{proper}} + W_{\texttt{improper}} * \mathcal{L}_{\texttt{improper}} \tag{4}$$

To prevent overfitting and ensure optimal model performance, we applied dropouts to the atom embedding stage (**Stage 1**) and symmetry-preserving stage (**Stage 2**), as well as implemented an early stopping mechanism. After 800 epochs, the joint root mean square error (RMSE) loss, which incorporates both energies and forces, was monitored using the validation set. This approach allowed us to identify the point at which further training no longer improved the model's generalization capability.

D Small molecule geometry optimization

The Python code used to benchmark the small molecule optimization geometries is available at https://github.com/choderalab/geometry-benchmark-espaloma, which is based on the OpenFF Infrastructures [37] used to validate and assess OpenFF 2.0.0 (Sage) [45].

[37] https://github.com/openforcefield/openff-sage/tree/main/inputs-and-results/benchmarks/qc-opt-geo

The QM-optimized conformer geometries and energies utilized in this study were obtained from `OpenFF Industry Benchmark Season 1 v1.1` [38] [89] deposited in QCArchive, which was generated at B3LYP-D3BJ/DZVP level of theory. This dataset consists nearly 9847 unique molecules and 76 713 conformers of drug-like molecules with mean molecular weight of 348 Da, and a maximum weight of 1104 Da. It includes formal charges of [-2, -1, 0, 1, 2] and covers atom elements of [Br, F, P, H, N, S, Cl, O, C]. The final benchmarking set consists 9728 unique molecules and 73 301 conformers, after filtering out connectivity changes during optimization, cases with stereochemistry which cannot be perceived, as well as any calculation failures due to convergence issues.

The QM-optimized molecules were minimized either with `espaloma-0.3`, `espaloma-0.3-rc1`, `openff-2.0.0`, `openff-2.1.0`, or `gaff-2.11` force fields using a L-BFGS optimizer implemented in OpenMM 8.0.0 [111] with a 5.0E-9 kJ/mol/nm convergence tolerance or maximum iteration set to 1500.

The MM-optimized molecules were assessed by measuring the root mean squared deviation (RMSD) in geometries between MM- and QM-optimized conformers, torsion fingerprint deviation (TFD), and error in relative conformer energies (ddE or $\Delta\Delta E$). The heavy atoms were used to superpose the MM- and QM-optimized molecules to compute the RMSD value using OpenEye Toolkits. TFD is a weighted metric of deviations in dihedral angles which overcomes the limitations of RMSD [133], which was computed using the RDKit package. $\Delta\Delta E$ is the energy difference between the MM and QM energies of conformer $\mathbf{x}_\mathrm{i}$, each with respect to the QM minimum energy conformer $\mathbf{x}_{0,\mathrm{QM}}$:

$$\Delta\Delta E_i = \Delta E_{\mathrm{MM,i}} - \Delta E_{\mathrm{QM,i}} = [E_{\mathrm{MM}}(\mathbf{x}_i) - E_{\mathrm{MM}}(\mathbf{x}_{0,\mathrm{QM}})] - [E_{\mathrm{QM}}(\mathbf{x}_i) - E_{\mathrm{QM}}(\mathbf{x}_{0,\mathrm{QM}})] \tag{5}$$

E MD simulations of peptides and folded proteins and calculation of NMR scalar couplings

All code used to setup, run, and analyze the peptide MD simulations—including experimental observables and model parameters—can be found at https://github.com/openforcefield/proteinbenchmark.

E.1 Peptides

A total of 121 experimental NMR observables are available for five homopeptides Ala_3, Ala_4, Ala_5, Gly_3, and Val_3 [91] as well as eight 3-mers Gly-X-Gly, where X is Ala, Glu, Phe, Lys, Leu, Met, Ser, or Val [92]. The initial structure for MD simulations was an extended conformation in which all backbone angles are $180°$, constructed from the amino acid sequence using the program `pmx` [134]. Protonation states were assigned at pH 2, consistent with the pH of the NMR experiments, using the PROPKA algorithm [135] in the program PDB2PQR 3.6.1 [136]. The peptides were then solvated in a rhombic dodecahedron of TIP3P water [26] with $1.4\,\mathrm{nm}$ padding and neutralizing sodium and chloride counterions using the Modeller module in OpenMM 8.0.0 [111]. Monovalent ions were modeled using parameters from Joung and Cheatham [29]. Force field parameters were assigned to the peptides using either Amber ff14SB [22] or `espaloma-0.3.2`, which is equivalent to `espaloma-0.3`. For `ff14SB`, RESP charges for Ala, Gly, and Val residues with protonated C termini were taken from Nerenberg and Head-Gordon [137]. Hydrogen mass repartitioning with a hydrogen mass of 3.0 amu was applied to the solutes. The solvated systems were energy minimized with Cartesian restraints applied to non-hydrogen solute atoms with an energy constant of $1.0\,\mathrm{kcal\,mol^{-1}}\,\mathrm{Å}^{-2}$.

MD simulations were performed using the CUDA platform of OpenMM 8.0.0 [111] with a Langevin Middle Integrator [138], a Monte Carlo barostat [139], and constraints on covalent hydrogen bond lengths. It is worth noting that increasing the time step beyond $2\,\mathrm{fs}$, solely using hydrogen bond constraints, potentially makes the simulation unstable. This leads to higher probability of instabilities as the simulation runs longer which is due to the limitations of the constraint algorithm itself. Both hydrogen mass repartitioning and hydrogen bond constraints are required for stable simulations to enable longer time steps [140].

The barostat equilibrium pressure was $1\,\mathrm{atm}$, and the thermostat equilibrium temperature was $300\,\mathrm{K}$ for the five homopeptides and $298\,\mathrm{K}$ for the eight Gly-X-Gly 3-mers. During a $1\,\mathrm{ns}$ equilibration period, the

[38] https://github.com/openforcefield/qca-dataset-submission/tree/master/submissions/2021-06-04-OpenFF-Industry-Benchmark-Season-1-v1.1

integrator time step was $1\,\mathrm{fs}$, the Langevin collision rate was $5\,\mathrm{ps}^{-1}$, and the barostat frequency was 5 steps. During a $500\,\mathrm{ns}$ production period, the integrator time step was $4\,\mathrm{fs}$, the Langevin collision rate was $1\,\mathrm{ps}^{-1}$, and the barostat frequency was 25 steps. Each peptide system and solute force field was simulated using three replicas.

Peptide backbone dihedral angles were extracted from trajectories using the program LOOS 4.0.4 [141]. $^3J_{\mathrm{HN,CA}}$ scalar couplings were estimated using parameters from Hennig et al. [105].

$$^3J_{\mathrm{HN,CA}}(\phi_i, \psi_{i-1}) = -0.23\cos\phi_i - 0.20\cos\psi_{i-1} + 0.07\sin\phi_i + 0.08\cos\psi_{i-1} + 0.07\cos\phi_i\cos\psi_{i-1} + 0.12\cos\phi_i\sin\psi_{i-1} - 0.08\sin\phi_i\cos\psi_{i-1} - 0.14\sin\phi_i\sin\psi_{i-1} + 0.54 \quad (6)$$

where ϕ_i is the ϕ dihedral angle for the current residue and ψ_{i-1} is the ψ dihedral angle for the previous residue. For all other scalar couplings, the scalar couplings were estimated using a Karplus model [104].

$$J(\theta) = A\cos^2(\theta + \Delta) + B\cos(\theta + \Delta) + C \quad (7)$$

where θ is the dihedral angle associated with the observable and A, B, C, and Δ are empirical Karplus parameters [98, 105–107] summarized in **SI Table 3**.

Agreement with experiment was quantitatively assessed by computing χ^2 values

$$\chi^2 = \frac{1}{N_{\mathrm{obs}}}\sum_{\mathrm{obs}}\frac{\left(J_{\mathrm{comp}} - J_{\mathrm{exp}}\right)^2}{\sigma^2_{\mathrm{model}}} \quad (8)$$

where the summation runs over observables, J_{comp} is the computed scalar coupling estimated using Eq. 7 or Eq. 6 averaged over all replicas, J_{exp} is the experimentally measured scalar coupling, and σ_{model} is the systematic error in the Karplus model, which is an order of magnitude larger than the uncertainty in the experimental values [91, 92]. The estimates of the systematic uncertainties in the Karplus models are provided in **SI Table 3**.

E.2 Folded proteins

System preparation and equilibration protocol was identical to the small peptides except that folded proteins were simulated using GROMACS 2022.5 [142] using both `ff14sb` and `espaloma-0.3`. The c-rescale barostat [139] was used along with the v-rescale thermostat [143] to establish an equilibrium temperature of $298\,\mathrm{K}$ and $1\,\mathrm{atm}$. Hydrogen mass repartitioning was applied with a hydrogen mass of 3.0 amu to enable the use of a time step of $4\,\mathrm{fs}$.

Backbone and χ_1 side chain ^3J-scalar couplings were estimated using the Karplus model as described in Eq. 7, similarly to those for peptides, with additional Karplus parameters for the side chains (**SI Table 3**). Additionally, inter-residue $^3J_{N,C'}$ scalar couplings for backbone hydrogen bonds were computed using Eq.12 of Barfield [109].

$$^3J_{N,C'}(R,\theta,\varphi) = \exp\left(-k\left(R - R_0\right)\right)\left(\left(A\cos^2\varphi + B\cos\varphi + C\right)\sin^2\theta + D\cos^2\theta\right) \quad (9)$$

where R is the distance between the amide hydrogen of the donor residue and the amide oxygen of the acceptor residue; θ is the angle between the donor hydrogen, the acceptor oxygen, and the acceptor carbon; and φ is the dihedral angle between the donor hydrogen, acceptor oxygen, acceptor carbon, and acceptor nitrogen. The other parameters in this expression taken from Barfield [109] are: k is $3.2\,\text{Å}^{-1}$, R_0 is $1.76\,\text{Å}$, A is 0.62 Hz, B is 0.92 Hz, C is 0.14 Hz, and D is -1.31 Hz. The estimates of the systematic uncertainties for inter-residue hydrogen bond scalar couplings in the Karplus models are provided in **SI Table 3**.

The conformational rigidity of residues in folded proteins leads to significant variance in the range of scalar coupling values (i.e., dihedral angles) across different coupling types. In contrast, each residue in short peptides samples essentially all available conformers, and the measured scalar coupling is an average over these populations, which does not vary much with sequence position or residue identity. Additionally, experimental scalar couplings can lie outside the range of the relevant Karplus curves, posing a challenge to

the reproduction of the experimental observations, regardless of the conformational ensembles sampled in simulations. To address this issue, the agreement with experimental data was quantitatively assessed by computing the average normalized error (ANE) values, rather than utilizing χ^2 values, as described by Maier et al. [22].

$$\text{ANE} = \frac{1}{N_{\text{obs}}} \sum_{\text{obs}} \frac{|J_{\text{comp}} - J^*_{\text{exp}}|}{\max\left(J_{\text{obs,Karplus}}\right) - \min\left(J_{\text{obs,Karplus}}\right)} \tag{10}$$

where J^*_{exp} is given by:

$$J^*_{\text{exp}} = \begin{cases} \min(J_{\text{obs,Karplus}})^3, & J_{\text{exp}} < \min(J_{\text{obs,Karplus}}) \\ \max(J_{\text{obs,Karplus}})^3, & J_{\text{exp}} > \max(J_{\text{obs,Karplus}}) \\ J^3_{\text{exp}}, & \text{otherwise} \end{cases} \tag{11}$$

Here, the deviations $|J_{\text{comp}} - J^*_{\text{exp}}|$ are normalized by the magnitude of the Karplus curve range. The experimental scalar couplings are adjusted to the values on the Karplus curve that lie closest if they fall outside the range; otherwise, the experimental value is used as the target. ANE value ranges from 0 to 1, where 0 indicates the best possible agreement and 1 indicates the maximum deviation.

F Protein-ligand benchmark dataset

The protein-ligand benchmark dataset can be found at https://github.com/kntkb/protein-ligand-benchmark-custom. It consists of 4 target systems (Tyk2, Cdk2, P38, and Mcl1) and a total of 76 ligands. This dataset was curated from the openforcefield/protein-ligand-benchmark repository (https://github.com/openforcefield/protein-ligand-benchmark/tree/d3387602bbeb0167abf00dfb81753d8936775dd2). Note that one of the ligand from P38 (`ligand_p38a_2ff`) was excluded from the dataset because of its ambigous stereochemistry. The protein structures and ligand poses, as well as the ligand transformations, were manually curated, while the experimental results were adopted from the original repository. The protein and ligand structures were prepared using Maestro from Schrodinger 2022-2.

The PDB structure of a protein-ligand complex was imported and processed using the default settings of `prepwizard`, along with additional options including filling in missing side chains and loops using Prime, capping termini, and deleting waters beyond 5.0 Å from het groups. The tautomer states of the ligand complexed with the protein were manually inspected, and the most reasonable state was chosen from a human perspective. For the protein residues, the protonation and tautomer states were optimized using the default settings of `H-bond assignment`. Subsequently, a restrained minimization was performed using the OPLS4 force field, with an RMSD convergence threshold of 0.3 Å for the heavy atoms. The minimized protein structure from the complex served as the initial protein structure, and X-ray water molecules were retained if necessary, such as buried water molecules in the binding pocket.

For the ligand poses, a flexible ligand alignment approach was applied with respect to the PDB ligand pose found in the protein-ligand complex structure. The default settings of `ligprep` were used to generate all possible ligand tautomer states, which were then visually inspected to choose the most reasonable state. Subsequently, ligand alignment was performed by aligning all ligands to the PDB ligand pose found in the protein-ligand complex structure, using the `Ligand Alignment` module in Maestro with Bemis-Murcko scaffold or maximum common scaffold constrain. The ligand poses were manually adjusted, taking into account the binding site environment, which involved rotating ligand torsions and minimizing selected atoms to alleviate severe atom clashes and obtain better initial poses.

Finally, the ligand transformation networks were defined manually by human experts, creating a outward radial map with the simplest ligand in the center. In the case of P38 and Mcl1, R-group substituent from multiple scaffold positions and scaffold hopping were observed. In such cases, ligand transformations were grouped into categories to resemble different structure-activity relationship purposes while maintaining a simplified ligand transformation network.

G Alchemical free energy calculations using protein-ligand benchmark dataset

The Python code used to perform the alchemical protein-ligand binding free energy benchmark experiment is available at https://github.com/choderalab/pl-benchmark-espaloma-experiment. We utilized the Perses 0.10.1 relative alchemical free energy calculation infrastructure [110], which is based on OpenMM 8.0.0 [111], openmmtools 0.22.1 [144], and a modified version of openmmforcefields 0.11.0 package [127] (https://github.com/kntkb/openmmforcefields/tree/6d2c3dcd33d9800a32032d28b6b2dca92f348a43) to support `espaloam-0.3`.

All systems were solvated with TIP3P water [26] with 9.0 Å buffer around the protein, and the system was neutralized with the Joung and Cheatham monovalent counterions [29] with 300 mM NaCl salt concentration. The protein was parametrized with Amber ff14SB force field [22], and the small molecules were parametrized with `openff-2.1.0` [83], `espaloma-0.3`, or `espaloma-0.2.2` [49]. Additionally, the protein-ligand was self-consistently parametrized with `espaloma-0.3`, and a modified version of Perses 0.10.1 (https://github.com/kntkb/perses/tree/0d069fc1cf31b8cce1ae7a1482c3fa46bc1382d2) was used to perform the protein-ligand binding free energy calculations.

Alchemical free energy calculations were simulated with replica exchange among Hamiltonians with Gibbs sampling [145]. All simulations were performed with 12 alchemical states for 10 ns/replica for Tyk2 and Cdk2, 15 ns/replica for Mcl1, and 20 ns/replica for P38, with replica exchange attempts made every 1 ps. The simulations were performed at 300 K and 1 atm using a Monte Carlo Barostat [139] and Langevin BAOAB integrator [146] with a collision rate of 1/ps. Bonds to hydrogen were constrained, and hydrogen atom masses were set to 3.0 amu by transferring the masses connected to the heavy atoms, allowing for simulations with a 4 fs timestep.

Atom mappings were generated from the provided geometries in the curated benchmark set (see **SI Section F**). Atoms within 0.5 Å of the transforming ligand pairs were detected as valid mapping atoms using the `use_given_geometries` functionality in Perses.

PyMBAR 3.1.1 [147] was used to compute the relative free energy, while absolute free energies up to an additive constant were estimated using a least-squares estimation strategy [148] using a modified version of OpenFE cinnabar 0.3.0 package [128] (https://github.com/kntkb/cinnabar/tree/de7bc6623fb25d75848aa1c9f538b77cd02a4b01). Both experimental and calculated absolute free energies were shifted to their respective means before computing the statistics.

H Tyk2 protein-ligand complex MD simulations

The unbiased MD simulation code used in this study, along with initial prepared structures, can be found at https://github.com/choderalab/vanilla-espaloma-experiment. The initial structures of Tyk2 and ligand #1 shown in **SI Figure13** was taken from the protein-ligand benchmark dataset as described in **SI Section F**. Two protein-ligand complex MD simulations were performed using `espaloma-0.3` to self-consistently parametrize both the protein and ligand, and `openff-2.1.0` and Amber ff14SB to parametrize the ligand and protein, respectively. Both systems were solvated with TIP3P water [26] and neutralized with the Joung and Cheatham monovalent counterions [29] with 150 mM NaCl salt concentration.

All simulations were performed at 300 K and 1 atm using a Monte Carlo Barostat [139] and Langevin Middle Integrator (a variant splitting of the BAOAB integrator) [138] with a collision rate of 1/ps. Bonds to hydrogen were constrained, and hydrogen atom masses were set to 3.0 amu allowing for simulations with a 4 fs timestep. The solvated systems were minimized and subsequently subjected to 3 microsecond of simulation using OpenMM 8.0.0 [111].

The root-mean square deviation (RMSD) profile of the heavy ligand atoms and protein Cα atoms were reported over the 3 microsecond MD simulation. The trajectories were aligned with respect to the binding pocket residues (within 4 Å from the initial ligand pose) before computing the heavy ligand atom RMSD. Similarly, the protein Cα atoms excluding the first and last 5 residues, were used to align the protein trajectories before the Cα RMSD calculation, with the first and last 5 residues excluded from the RMSD computation. The root-mean-square fluctuation (RMSF) profile of the protein Cα atoms was computed relative to the averaged structure. The trajectories were aligned to the Cα atoms, with the first and last five residues excluded,

before the RMSF calculation was performed.

It is worth noting that the experimental RMSF can be related to isotropic B-factors found in X-ray crystal structures using the following relation:

$$B = \frac{8\pi^2}{3}\mathrm{RMSF}^2 \tag{12}$$

Although a crystal structure bound with ligand #1 was not available, we compared the experimental and computed RMSF derived from simulations to address whether the experimental peaks are recapitulated in the simulations. The experimental RMSF was derived from the isotropic B-factors of the Tyk2 X-ray crystal structure (PDB ID: 4GIH) complexed with ligand #8, as shown in **SI Figure13**, which is topologically highly similar to ligand #1.

The overall trend of simulated RMSF peaks is similar to experimental RMSF peaks, which were computed from the isotropic B-factors of the Tyk2 X-ray crystal structure (PDB ID: 4GIH) used as the initial protein structure for the MD simulation. The Pearson correlations are 0.58 and 0.70 for `espaloma-0.3` and `ff14SB+openff-2.1.0`, respectively. However, the RMSF peaks derived from simulations are higher in flexible regions and underestimated in less flexible regions. This outcome is unsurprising and expected, as the B-factors include contributions from various sources, such as vibration and static disorder, lattice defects, and crystal packing effects [149, 150].

Dataset (QCArchive Workflow)	Category	Mols	Confs	Split	**espaloma-0.3** Energy RMSE (kcal/mol) Force RMSE (kcal/mol · $Å^{-1}$)			**Repetition** Energy RMSE (kcal/mol) Force RMSE (kcal/mol · $Å^{-1}$)		
					Train (80%)	Validate (10%)	Test (10%)	Train (80%)	Validate (10%)	Test (10%)
**SPICE-Pubchem [71, 84]**<br>(Dataset)	Small molecule	14110	608436	80:10:10	$2.06_{2.04}^{2.07}$<br>$6.22_{6.19}^{6.26}$	$2.31_{2.25}^{2.37}$<br>$6.79_{6.65}^{6.95}$	$2.30_{2.25}^{2.36}$<br>$6.81_{6.68}^{6.95}$	$2.01_{1.99}^{2.03}$<br>$6.18_{6.15}^{6.21}$	$2.23_{2.19}^{2.28}$<br>$6.73_{6.57}^{6.94}$	$2.25_{2.20}^{2.30}$<br>$6.64_{6.51}^{6.78}$
**SPICE-DES-Monomers [71, 73]**<br>(Dataset)	Small molecule	369	18435	80:10:10	$1.39_{1.32}^{1.46}$<br>$5.86_{5.69}^{6.02}$	$1.34_{1.13}^{1.60}$<br>$5.63_{5.12}^{6.24}$	$1.36_{1.13}^{1.67}$<br>$5.91_{5.49}^{6.42}$	$1.36_{1.29}^{1.43}$<br>$5.83_{5.66}^{5.99}$	$1.38_{1.13}^{1.68}$<br>$5.56_{5.24}^{5.96}$	$1.41_{1.20}^{1.64}$<br>$5.92_{5.42}^{6.57}$
**Gen2-Opt**<br>(OptimizationDataset)	Small molecule	1024	244989	80:10:10	$1.36_{1.26}^{1.48}$<br>$3.94_{3.79}^{4.11}$	$1.35_{1.17}^{1.56}$<br>$4.22_{3.92}^{4.52}$	$1.66_{1.21}^{2.29}$<br>$4.47_{3.90}^{5.40}$	$1.31_{1.20}^{1.43}$<br>$3.77_{3.64}^{3.92}$	$1.51_{1.15}^{1.93}$<br>$4.76_{3.91}^{6.01}$	$1.41_{1.16}^{1.71}$<br>$4.32_{3.71}^{5.09}$
**Gen2-Torsion**<br>(TorsionDriveDataset)	Small molecule	729	25832	80:10:10	$1.76_{1.61}^{1.91}$<br>$4.31_{4.18}^{4.44}$	$1.97_{1.60}^{2.42}$<br>$5.00_{4.49}^{5.55}$	$1.64_{1.32}^{2.01}$<br>$4.71_{4.18}^{5.29}$	$1.66_{1.52}^{1.79}$<br>$4.25_{4.12}^{4.38}$	$1.91_{1.48}^{2.37}$<br>$4.56_{4.12}^{5.01}$	$1.84_{1.43}^{2.26}$<br>$5.40_{4.26}^{7.03}$
**SPICE-Dipeptide [71]**<br>(Dataset)	Peptide	677	26279	80:10:10	$3.21_{3.16}^{3.26}$<br>$7.98_{7.88}^{8.07}$	$3.15_{3.01}^{3.30}$<br>$8.05_{7.77}^{8.34}$	$3.09_{2.96}^{3.21}$<br>$7.78_{7.55}^{8.02}$	$3.06_{3.01}^{3.11}$<br>$7.81_{7.71}^{7.90}$	$3.15_{3.02}^{3.29}$<br>$7.74_{7.47}^{7.97}$	$2.94_{2.82}^{3.07}$<br>$7.64_{7.39}^{7.87}$
**Pepconf-Opt [74]**<br>(OptimizationDataset)	Peptide	557	166291	80:10:10	$2.61_{2.43}^{2.83}$<br>$3.83_{3.60}^{4.09}$	$2.82_{2.41}^{3.27}$<br>$3.65_{3.29}^{4.12}$	$2.79_{2.45}^{3.13}$<br>$4.01_{3.63}^{4.46}$	$2.56_{2.40}^{2.73}$<br>$3.78_{3.58}^{4.02}$	$2.87_{2.24}^{3.77}$<br>$3.92_{3.43}^{4.62}$	$3.20_{2.45}^{4.17}$<br>$4.29_{3.53}^{5.49}$
**Protein-Torsion**<br>(TorsionDriveDataset)	Peptide	62	48999	80:10:10	$2.27_{2.06}^{2.50}$<br>$3.94_{3.70}^{4.24}$	$1.91_{1.36}^{2.28}$<br>$3.49_{2.85}^{3.97}$	$1.93_{1.73}^{2.14}$<br>$3.49_{3.22}^{3.78}$	$2.20_{2.02}^{2.39}$<br>$3.85_{3.56}^{4.19}$	$2.52_{1.85}^{3.16}$<br>$4.21_{3.65}^{5.00}$	$2.46_{1.80}^{3.40}$<br>$4.01_{3.55}^{4.62}$
**RNA-Diverse**<br>(Dataset)	RNA	64	3703	80:10:10	$4.12_{3.95}^{4.31}$<br>$4.44_{4.40}^{4.47}$	$4.51_{4.05}^{4.92}$<br>$4.54_{4.50}^{4.58}$	$4.17_{3.85}^{4.52}$<br>$4.41_{4.29}^{4.51}$	$4.13_{3.95}^{4.29}$<br>$4.42_{4.39}^{4.46}$	$4.57_{4.04}^{5.18}$<br>$4.54_{4.50}^{4.59}$	$4.12_{3.68}^{4.71}$<br>$4.47_{4.39}^{4.54}$
**RNA-Trinucleotide**<br>(Dataset)	RNA	64	35811	0:0:100	—<br>—	—<br>—	$3.75_{3.59}^{3.94}$<br>$4.28_{4.20}^{4.39}$	—<br>—	—<br>—	$3.80_{3.64}^{3.97}$<br>$4.27_{4.20}^{4.37}$
**RNA-Nucleoside**<br>(Dataset)	RNA	4	9542	100:0:0	$1.32_{1.16}^{1.49}$<br>$4.17_{3.86}^{4.47}$	—<br>—	—<br>—	$1.26_{1.11}^{1.43}$<br>$4.00_{3.67}^{4.33}$	—<br>—	—<br>—

Table S 1. A repeated Espaloma refitting experiment yields consistent results with `espaloma-0.3`, capable of accurately fitting quantum chemical energies and forces. The Espaloma refitting experiment was conducted using a different random seed to partition the datasets into train, validate, and test sets. The RMSE metrics of energy and forces were analyzed similarly to those of `espaloma-0.3`. The 95% confidence intervals, annotated in the results, were calculated by bootstrapping molecule replacement using 1000 replicates.

		GB3	BPTI	Lysozyme	Ubiquitin
χ^2	`ff14sb`	4.63 ± 0.62	21.80 ± 6.43	37.97 ± 11.40	12.04 ± 0.84
	`espaloma-0.3`	12.96 ± 1.76	27.64 ± 6.09	54.45 ± 9.66	17.25 ± 2.65
χ^2_{BB}	`ff14sb`	5.19 ± 0.75	10.65 ± 2.87	—	8.71 ± 0.90
	`espaloma-0.3`	10.81 ± 1.38	12.90 ± 2.79	—	9.19 ± 1.05
χ^2_{SC}	`ff14sb`	3.77 ± 1.23	30.05 ± 10.84	37.97 ± 9.4	14.75 ± 3.65
	`espaloma-0.3`	16.27 ± 3.91	38.54 ± 10.10	54.45 ± 9.66	24.08 ± 4.76
ANE	`ff14sb`	0.091 ± 0.005	0.151 ± 0.019	0.219 ± 0.023	0.126 ± 0.007
	`espaloma-0.3`	0.156 ± 0.009	0.183 ± 0.019	0.282 ± 0.024	0.147 ± 0.008
$\mathrm{ANE}_{\mathrm{BB}}$	`ff14sb`	0.096 ± 0.006	0.119 ± 0.017	—	0.132 ± 0.007
	`espaloma-0.3`	0.143 ± 0.009	0.133 ± 0.018	—	0.133 ± 0.007
$\mathrm{ANE}_{\mathrm{SC}}$	`ff14sb`	0.084 ± 0.010	0.176 ± 0.030	0.219 ± 0.023	0.121 ± 0.017
	`espaloma-0.3`	0.176 ± 0.066	0.220 ± 0.030	0.282 ± 0.024	0.159 ± 0.014

Table S 2. The χ^2 and absolute normalized error (ANE) values quantifying the deviations of simulated NMR scalar couplings compared to experimental measurements for GB3, BPTI, lysozyme, and ubiquitin using `ff14sb` and `espaloma-0.3`. χ^2 and ANE values are reported for the entire protein, as well as for the backbone (BB) and side chain (SC) regions. The mean and 95% CI for each metric are reported from the critical values of a Student's t-distribution based on three replicates of the 10 μs simulations.

Observable	θ	Δ	A	B	C	σ_{model}	System	Reference
**Backbone**								
$^{1}J_{\text{N,CA}}$	ψ_i	0.0	1.70	−0.98	9.51	0.59	peptide	Wirmer and Schwalbe [106]
$^{2}J_{\text{N,CA}}$	ψ_{i-1}	0.0	−0.66	−1.52	7.85	0.50	peptide	Ding and Gronenborn [107]
$^{3}J_{\text{HA,C}'}$	ϕ_i	120.0	3.72	−2.18	1.28	0.38[a]	peptide, protein	Hu and Bax [102]
$^{3}J_{\text{HN,CB}}$	ϕ_i	60.0	3.51	−0.53	0.14	0.25	peptide, protein	Vögeli et al. [98]
$^{3}J_{\text{HN,C}'}$	ϕ_i	180.0	4.12	−1.10	0.11	0.31	peptide, protein	Vögeli et al. [98]
$^{3}J_{\text{HN,HA}}$	ϕ_i	−60.0	7.97	−1.26	0.63	0.42	peptide, protein	Vögeli et al. [98]
$^{3}J_{\text{HN,CA}}$	ϕ_i, ψ_{i-1}		Eq. 6			0.10	peptide	Hennig et al. [105]
**Side chain**								
$^{3}J_{\text{C}',\text{CG1}}$(Val)	χ_1	−115.0	3.42	−0.59	0.17	0.25	protein	Chou et al. [96]
$^{3}J_{\text{C}',\text{CG2}}$(Ile)	χ_1	125.0	3.42	−0.59	0.17	0.25	protein	Chou et al. [96]
$^{3}J_{\text{C}',\text{CG2}}$(Thr)	χ_1	137.0	2.76	−0.67	0.19	0.21	protein	Chou et al. [96]
$^{3}J_{\text{C}',\text{CG2}}$(Val)	χ_1	5.0	3.42	−0.59	0.17	0.25	protein	Chou et al. [96]
$^{3}J_{\text{N,CG1}}$(Val)	χ_1	6.0	2.64	0.26	−0.22	0.25	protein	Chou et al. [96]
$^{3}J_{\text{N,CG2}}$(Ile)	χ_1	−114.0	2.64	0.26	−0.22	0.25	protein	Chou et al. [96]
$^{3}J_{\text{N,CG2}}$(Thr)	χ_1	−113.0	2.01	0.21	−0.12	0.21	protein	Chou et al. [96]
$^{3}J_{\text{N,CG2}}$(Val)	χ_1	126.0	2.64	0.26	−0.22	0.25	protein	Chou et al. [96]
$^{3}J_{\text{HA,HB}}$(Ile, Val)	χ_1	0.0	7.23	−1.37	1.79	0.40	protein	Pérez et al. [108]
$^{3}J_{\text{HA,HB}}$(Thr)	χ_1	0.0	7.23	−1.37	0.81	0.40	protein	Pérez et al. [108]
$^{3}J_{\text{HA,HB2}}$	χ_1	−120.0	7.23	−1.37	2.40	0.40	protein	Pérez et al. [108]
$^{3}J_{\text{HA,HB2}}$(Cys)	χ_1	−120.0	7.23	−1.37	1.71	0.40	protein	Pérez et al. [108]
$^{3}J_{\text{HA,HB2}}$(Ser)	χ_1	−120.0	7.23	−1.37	1.42	0.40	protein	Pérez et al. [108]
$^{3}J_{\text{HA,HB3}}$	χ_1	0.0	7.23	−1.37	2.40	0.40	protein	Pérez et al. [108]
$^{3}J_{\text{HA,HB3}}$(Cys)	χ_1	0.0	7.23	−1.37	1.71	0.40	protein	Pérez et al. [108]
$^{3}J_{\text{HA,HB3}}$(Ser)	χ_1	0.0	7.23	−1.37	1.42	0.40	protein	Pérez et al. [108]
**H-Bond**								
$^{3}J_{\text{N,C}'}$	H-bond		Eq. 9			0.12	protein	Barfield [109]

Table S 3. Karplus parameters used to estimate NMR scalar couplings. Empirical Karplus parameters Δ, A, B, and C used to estimate scalar couplings via Eq. 7 to estimate χ^2 and average normalized error (ANE) values via Eq. 8 and Eq. 10, respectively. The systematic errors in Karplus models σ to estimate χ^2 are also reported. [a]Systematic error estimate for the $^{3}J_{\text{HA,C}'}$ Karplus model taken from Wickstrom et al. [151]

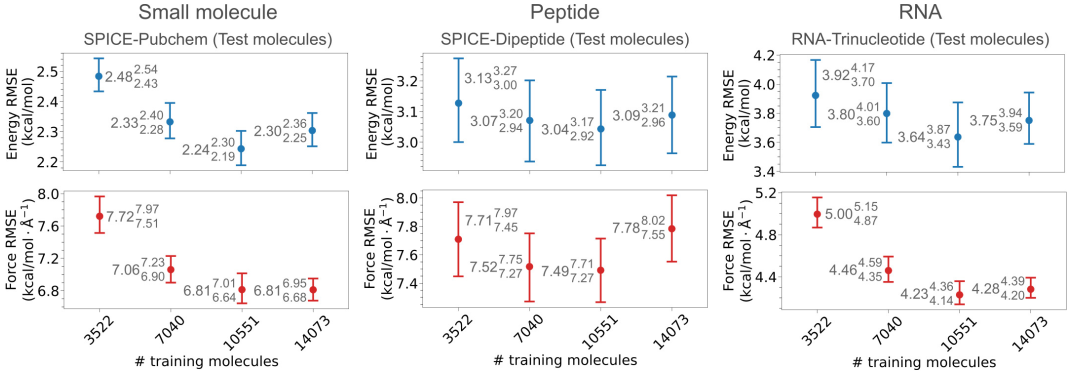

Figure S 1. Espaloma framework can directly fit to quantum chemical energies and forces even in low data regimes. The espaloma refitting experiment was conducted with a varying number of molecules in the training set. The same validation and test sets used to develop `espaloma-0.3` were maintained consistently throughout this experiment. The energy and force RMSE values on the test dataset are reported for the `SPICE-Pubchem`, `SPICE-Dipeptide`, and `RNA-Trinucleotide` datasets to illustrate the outcomes for small molecule, peptide, and RNA chemical series. The 95% confidence intervals, as annotated in the results, were calculated by bootstrapping molecule replacement using 1000 replicates.

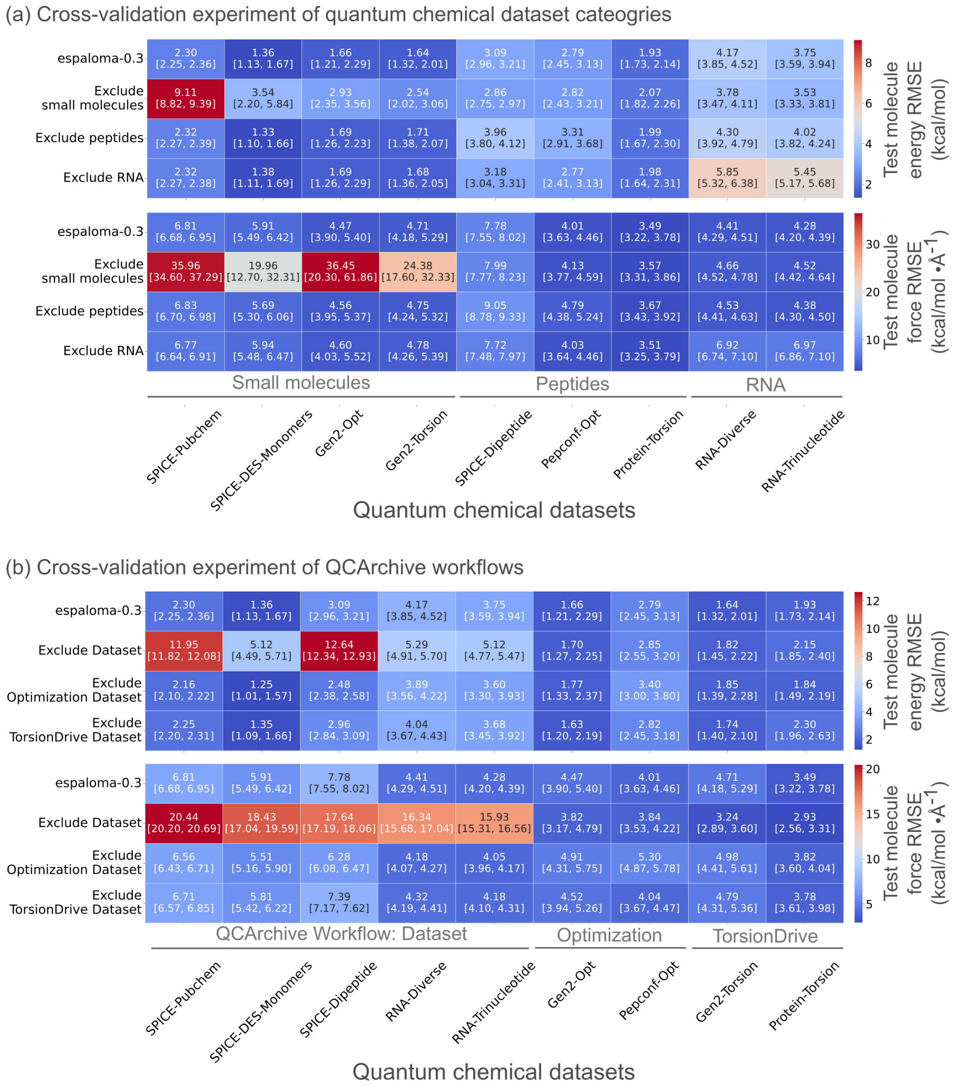

Figure S 2. Chemical diversity and high-energy conformers are important for accurately capturing quantum chemical energies and forces with Espaloma. Espaloma refitting experiments were conducted by excluding certain quantum chemical datasets during training and validation, following the procedures outlined in deploying `espaloma-0.3`. These experiments aimed to investigate how the quantum chemical datasets used for training espaloma affect its ability to accurately reproduce quantum chemical energies and forces. The refitting experiment was conducted with two different scenarios: (a) Quantum chemical datasets corresponding to the small molecules, peptides, or RNA chemical series were excluded from both training and validation; or (b) Quantum chemical datasets generated using the three distinct QCArchive workflows (see **SI Section B**) — `Dataset`, `Optimization Dataset`, or `TorsionDrive Dataset` — were excluded from both training and validation. The energy and force RMSE metrics for the test molecules, including the quantum chemical datasets excluded during training and validation, are reported with 95% confidence intervals. These intervals were calculated by bootstrapping molecule replacement with 1000 replicas and are depicted in square brackets.

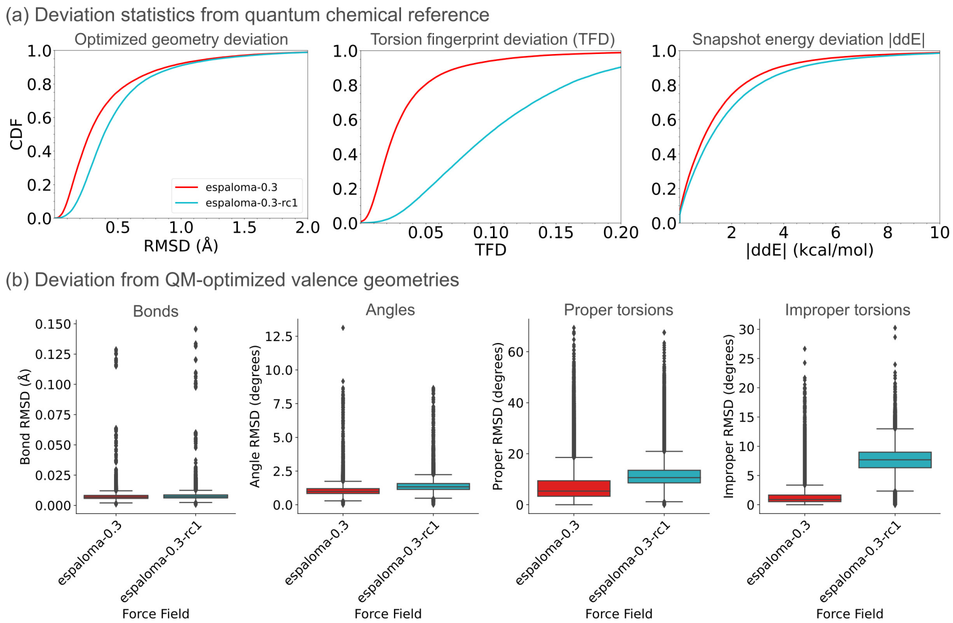

Figure S 3. Espaloma trained with regularizations against torsion terms can better preserve quantum chemical energy minima. A benchmark of gas-phase QM-optimized geometries, namely `OpenFF Industry Benchmark Season 1 v1.1` [89] from QCArchive, comprising nearly 9728 unique molecules and 73 301 conformers, was used to compare the structures and energetics of conformers optimized with `espaloma-0.3` and `espaloma-0.3-rc1` with respect to their QM-optimized goemetries at the B3LYP-D3BJ/DZVP level of theory. `espaloma-0.3-rc1` is a model created using the hyperparameters determined during its tuning process (see **Section C**), which does not apply any regularizations to torsion terms. (a) The cumulative distribution functions of root-mean-square deviation of atomic positions (RMSD), torsion fingerprint deviation (TFD) score, and relative energy differences (ddE) as described in a previous work [90] are reported. (b) Distributions of bond, angle, proper torsion, and improper torsion RMSD within each conformer with respect to its QM-optimized geometries are shown as quartile box plots. Lower values for all metrics indicate that the MM-optimized geometry is close to the quantum chemical reference structure.

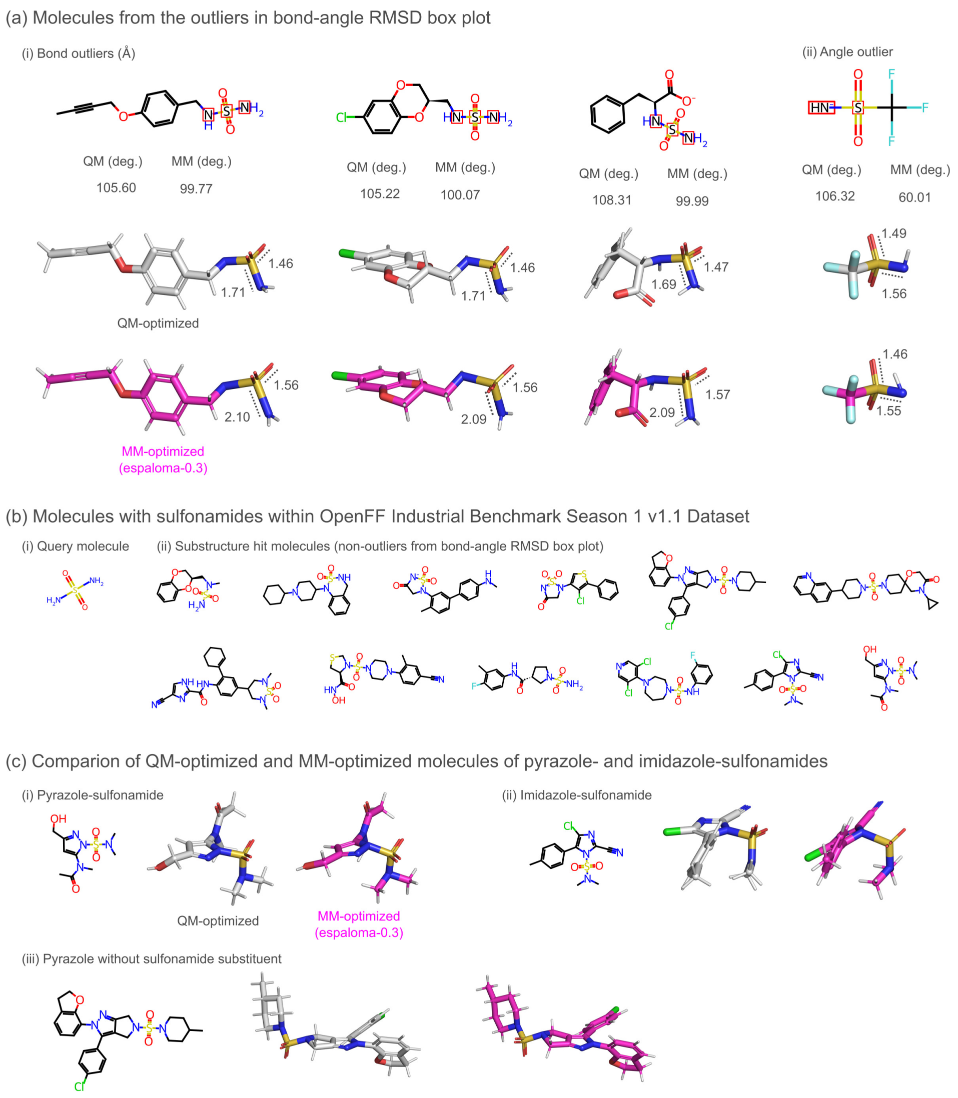

Figure S 4. Molecules containing sulfonamides are more challenging to maintain their QM-optimized geometries when minimized with espaloma-0.3 compared to other molecules. (a) Representative molecular conformers identified as outliers in the bond-angle RMSD box plot (**Figure 2**) are shown, where bond RMSD > 0.1 Å and angle RMSD > 10 degrees were considered as outliers. Three sulfonamide molecules connected to an aliphatic carbon exhibit elongated bond (S-O and S-N) distances respect to QM-optimized geometries, and a single angle outlier with a deviation of ∼40 degrees deviation from QM-optimized geometry was observed. (b) Molecules containing sulfonamide groups, excluding the outliers in (a) are shown, with each molecular conformer featuring reasonable bond distances within the sulfonamide group. (c) The nitrogen geometry of pyrazoles and imidazoles substituted with sulfonamides becomes trigonal pyramidal when minimized with `espaloma-0.3`, rather than preserving a flat ring geometry and losing their sp2 hybridized features, as observed with QM-optimized geometries.

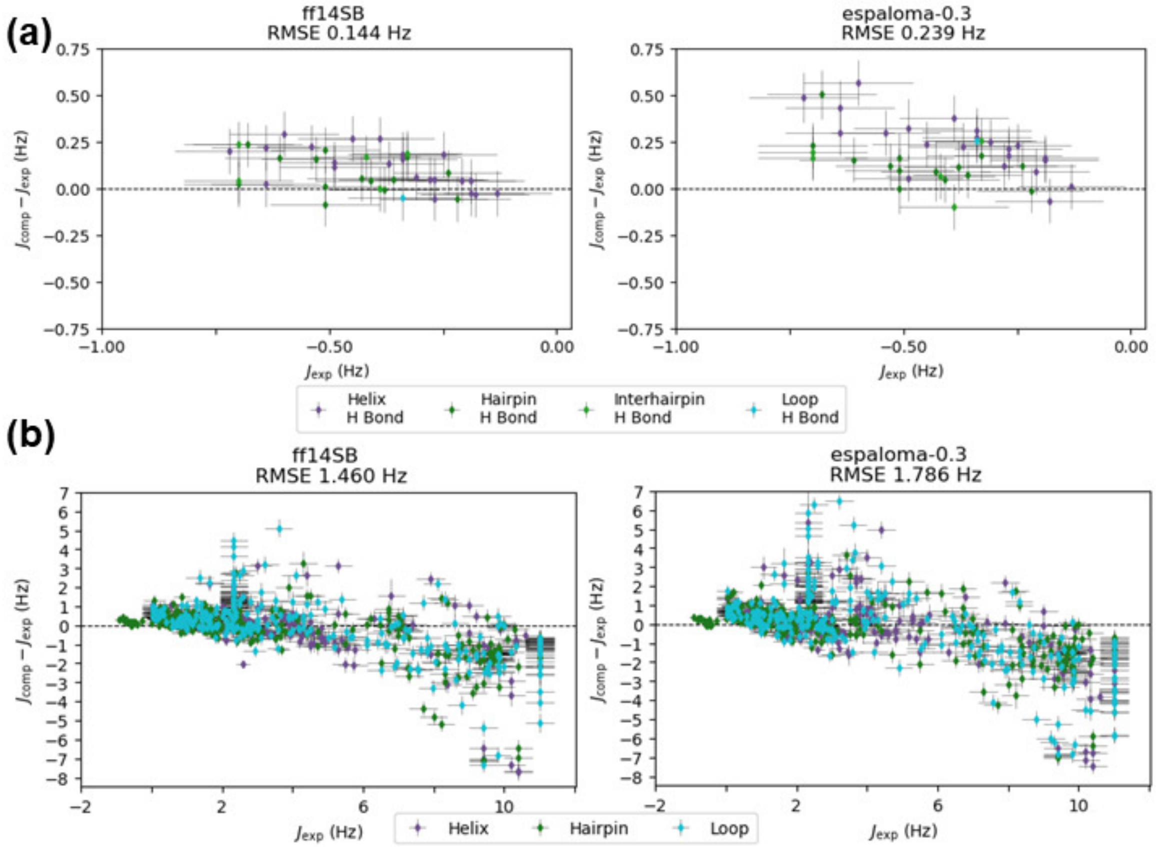

Figure S 5. `espaloma-0.3` reproduces NMR scalar couplings but slightly less accurately than the well-established biomolecular force field, `ff14sb`. The experimental and computed NMR scalar couplings between `ff14sb` and `espaloma-0.3` are compared for (a) backbone hydrogen bond (H-bond) scalar couplings in the helix, hairpin, interhairpin, and loop regions, which includes both the backbone and side chains, and (b) backbone scalar couplings from the helix, hairpin, and loop regions. Colors indicate the identity of secondary structures associated with each scalar coupling. Horizontal error bars represent the estimate of the systematic error in the experimental scalar coupling, and vertical error bars represent the uncertainty due to the computed estimate (standard error of the mean across 3 replicates) and the uncertainty due to the experimental value (systematic error) added in quadrature.

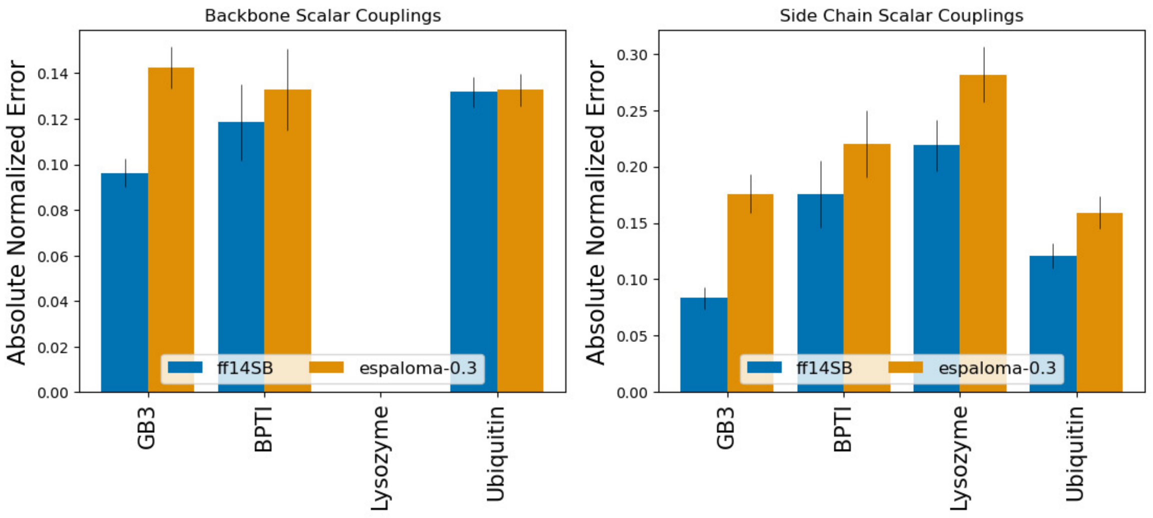

Figure S 6. `espaloma-0.3` reproduces experimental NMR scalar couplings of folded globular proteins with a slightly higher error than the well-established biomolecular force field, `ff14sb`, particularly for the side chains. The absolute normalized error (ANE) values, quantifying the deviations of simulated NMR scalar couplings from 10 μs trajectories compared to experimental measurements for GB3, BPTI, lysozyme, and ubiquitin, are depicted for both the backbone and side chain regions. For lysozyme, the column for backbone scalar couplings is not reported, as there were no backbone scalar coupling measurements in the dataset used for this study. Error bars represent a 95% confidence interval, constructed from the critical values of a Student's t-distribution and the standard error of the mean across the NMR observables, based on three replicates of the 10 μs simulation.

(a) Fraction of backbone categories

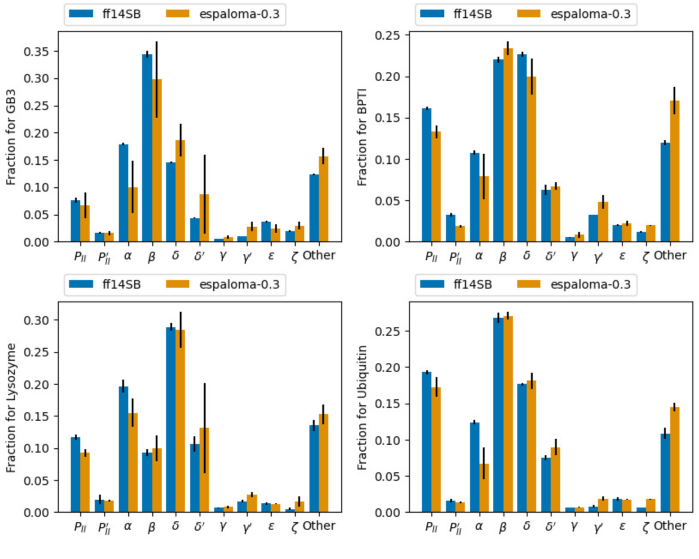

(b) Reference Ramachandran plot defining the backbone categories

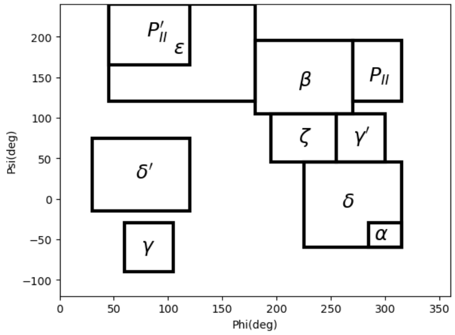

Figure S 7. Simulations with `espaloma-0.3` tend to show a greater decrease in the occupancy of defined folded regions, such as the alpha (α) and beta (β) backbone structures, compared to `ff14sb`. The populations of different dihedral clusters, based on Ramachandran angles, were compared. (a) Clusters of backbone dihedral angles simulated with `espaloma-0.3` and `ff14sb` are depicted for GB3, BPTI, lysozyme, and ubiquitin. In general, occupancy in defined folded regions (e.g., α and β) decreases, while occupancy in non-defined 'other' regions representing any dihedral pair outside the defined clusters, which are likely to be disordered. Error bars represent a 95% confidence interval, constructed from the critical values of a Student's t-distribution and the standard error of the mean across the NMR observables, based on three replicates of the 10 μs simulation. (b) A Ramachandran plot is shown to define the backbone dihedral angle clusters for clarity.

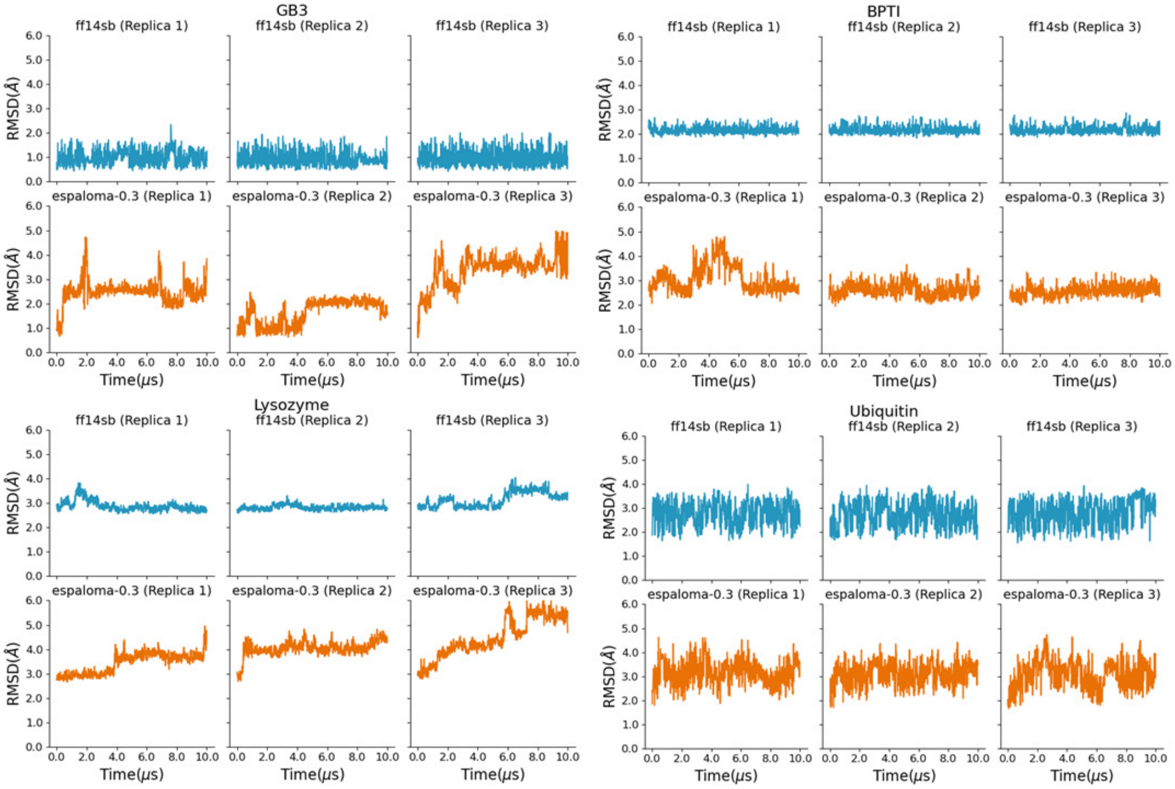

Figure S 8. `espaloma-0.3` exhibits slightly more backbone flexibility compared to `ff14sb`. The Cα RMSD for (a) GB3, (b) BPTI, (c) lysozyme, and (d) ubiquitin with respect to their initial PDB structures is computed.

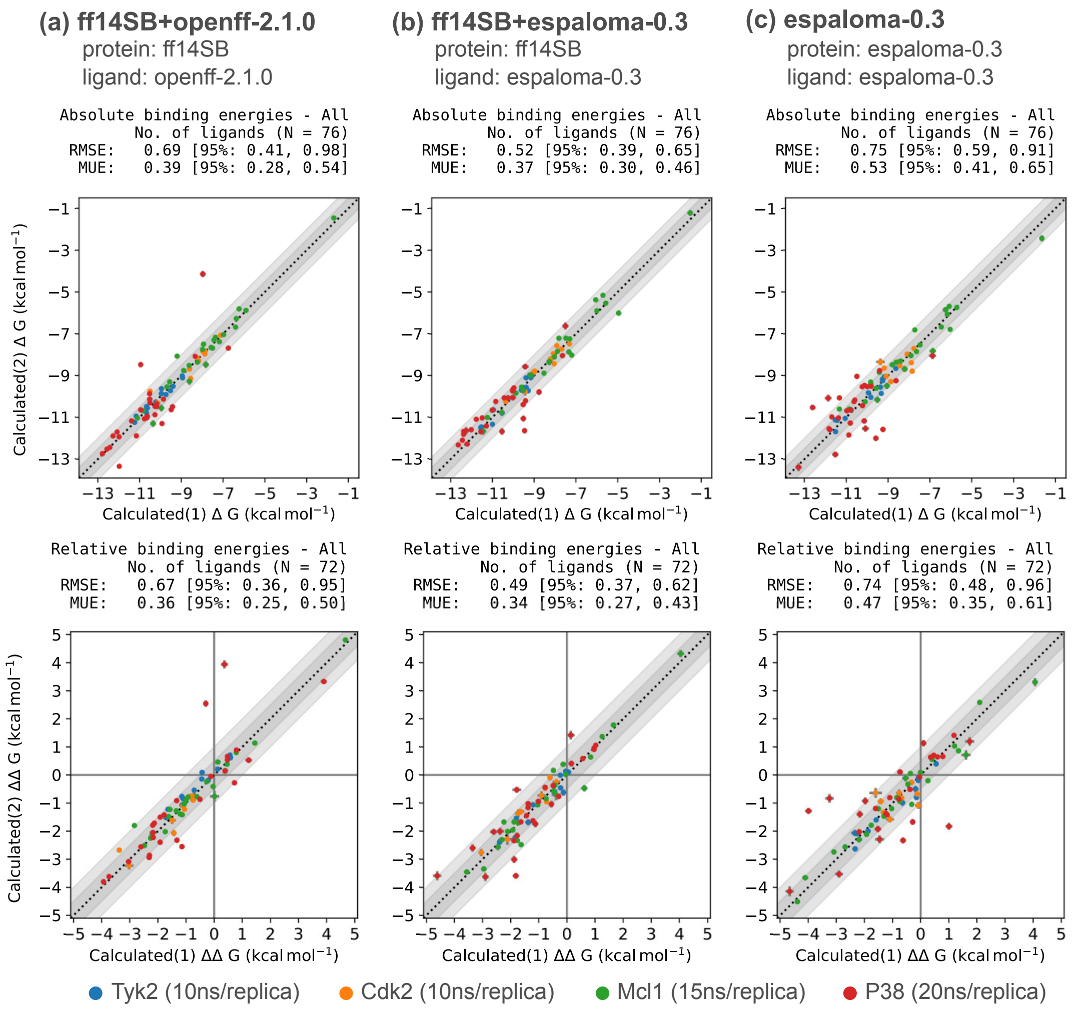

Figure S 9. Alchemical free energy calculations are well-reproduced within 10-20 ns of simulation time. The reproducibility of alchemical protein-ligand free energy calculations described in **Section 6** was investigated by conducting repeated simulations on Tyk2 (10 ns/replica), Cdk2 (10 ns/replica), Mcl1 (15 ns/replica), and P38 (20 ns/replica) using the same simulation protocols. The small molecules were parametrized either with (a) `openff-2.1.0`, (b) `espaloma-0.3` combined with Amber ff14SB for proteins, or (c) by paramterizing both small molecule and protein self-consistently with `espaloma-0.3`. The light and dark gray regions depict the confidence bounds of 0.5 kcal/mol and 1.0 kcal/mol, respectively.

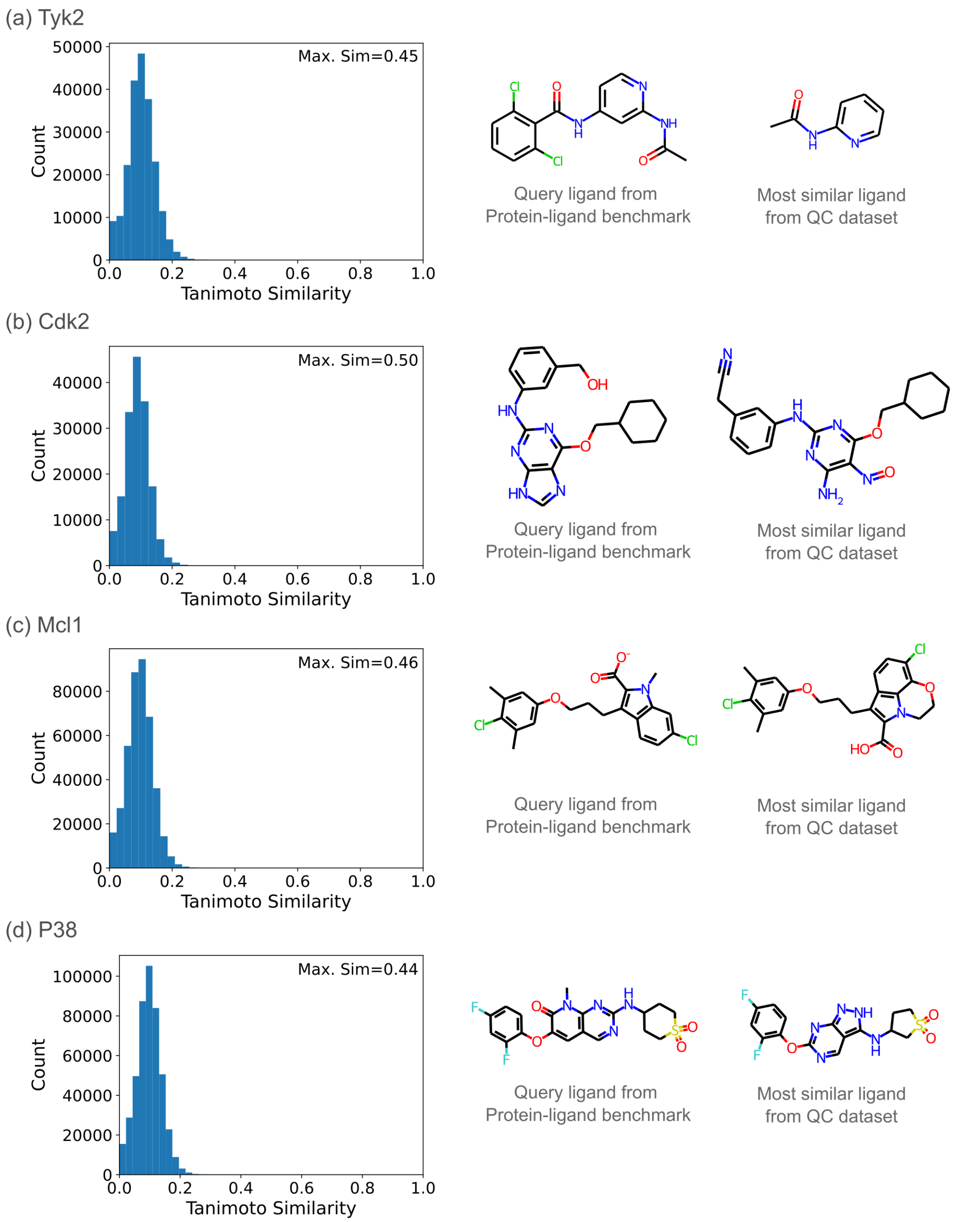

Figure S 10. The ligands from the protein-ligand binding free energy benchmark dataset significantly differ from the quantum chemical (QC) dataset used to train `espaloma-0.3`. Pairwise Tanimoto similarity scores between the ligands from the protein-ligand binding free energy benchmark dataset and the QC datasets used to deploy `espaloma-0.3` were investigated using Morgan Fingerprints implemented in RDKit [130], with a radius of 2 and a bit vector size of 2048. The maximum Tanimoto similarity score is reported for each target system in the protein-ligand binding free energy benchmark dataset, along with the molecular pair that achieved the maximum similarity score.

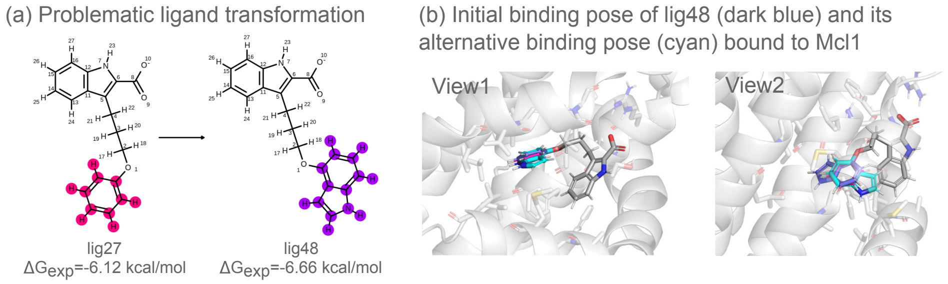

Figure S 11. The alchemical protein-ligand binding free energy calculation for the outlier Mcl1 ligand can be improved by adopting an alternative binding pose. (a) Illustration of the problematic Mcl1 ligand transformation observed as an outlier during the alchemical protein-ligand binding free energy calculation in **Figure 5**. The transforming ligand atoms are colored in magenta and purple. (b) The initial complex structure of Mcl1, bound with ligand #48 (in dark blue), used to simulate the alchemical free energy calculations, is illustrated along with its alternative flipped binding pose (in cyan).

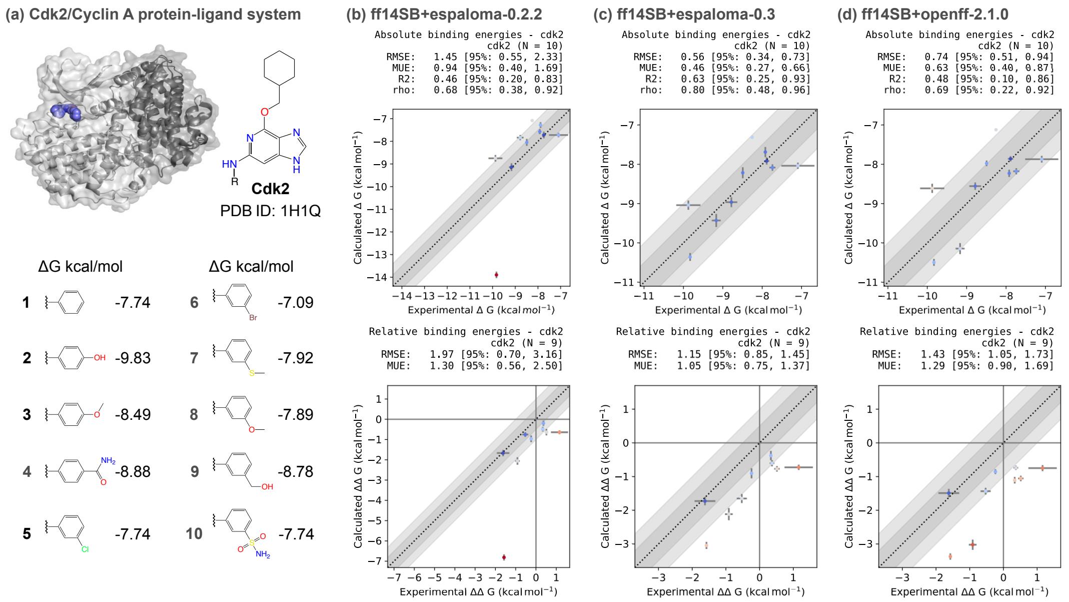

Figure S 12. Training `espaloma-0.3` on an extensive quantum chemical dataset significantly improves protein-ligand binding affinity calculations on the Cdk2 system. (a) We show the X-ray structure used for free energy calculation, along with the 2D structures of all ligands in the Cdk2 protein-ligand benchmark dataset. An outward radial map with ligand #1 in the center was used for the alchemical ligand transformations. We used the Perses 0.10.1 relative free energy calculation infrastructure [110] to calculate the relative free energy and assess the performance of (b) `espaloma-0.2.2` [49], (c) `espaloma-0.3`, and (d) `openff-2.1.0` [83] by parametrizing the small molecules with each force field. Amber ff14SB force field [22] was used to parametrize the protein for all cases. `espaloma-0.2.2` and `espaloma-0.3` achieves an absolute free energy (ΔG) RMSE of 1.45 [95% CI: 0.55, 2.33] kcal/mol and 0.56 [95% CI: 0.34, 0.73] kcal/mol, respectively, indicating that `espaloma-0.3` trained on extensive quantum chemical dataset significantly improved protein-ligand binding affinity calculations on the Cdk2 system.

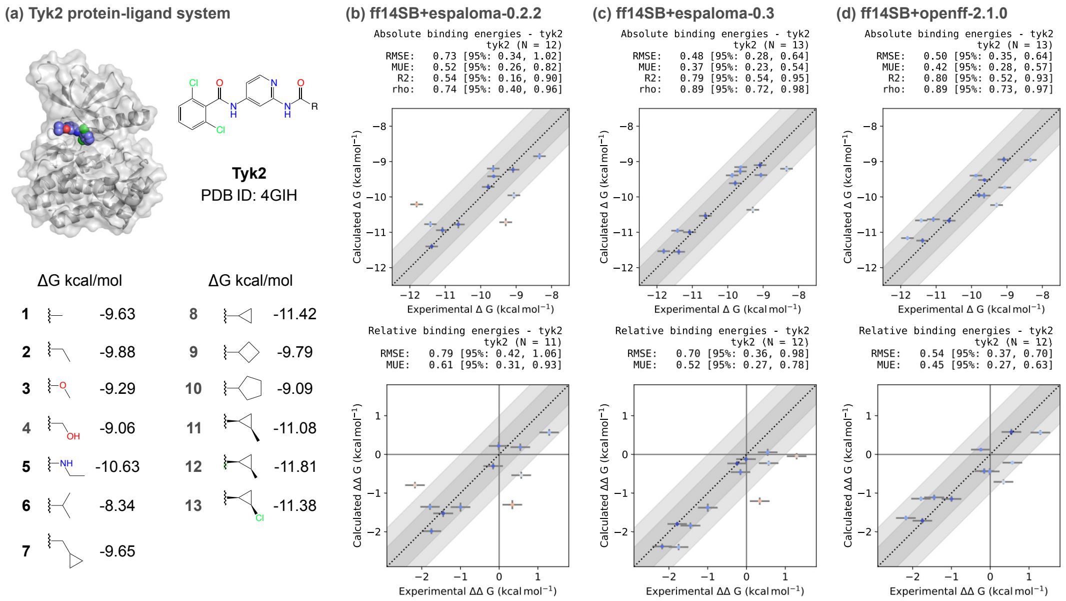

Figure S 13. Training `espaloma-0.3` on an expanded quantum chemical dataset improves protein-ligand binding affinity on the Tyk2 system. (a) We show the X-ray structure used for free energy calculation, along with the 2D structures of all ligands in the Tyk2 protein-ligand benchmark dataset. An outward radial map with ligand #1 in the center was used for the alchemical ligand transformations. We used the Perses 0.10.1 relative free energy calculation infrastructure [110] to calculate the relative free energy and assess the performance of (b) `espaloma-0.2.2` [49], (c) `espaloma-0.3`, and (d) `openff-2.1.0` [83] by parametrizing the small molecules with each force field. Amber ff14SB force field [22] was used to parametrize the protein for all cases. Notably, `espaloma-0.2.2` failed to simulate the alchemical ligand transformation of ligand #1 to ligand #2; hence one ligand is not reported in (b). `espaloma-0.2.2` and `espaloma-0.3` achieves an absolute free energy (ΔG) RMSE of 0.73 [95% CI: 0.34, 1.02] kcal/mol and 0.48 [95% CI: 0.28, 0.64] kcal/mol, respectively, suggesting that `espaloma-0.3` tends to show improved performance over `espaloma-0.2.2`.

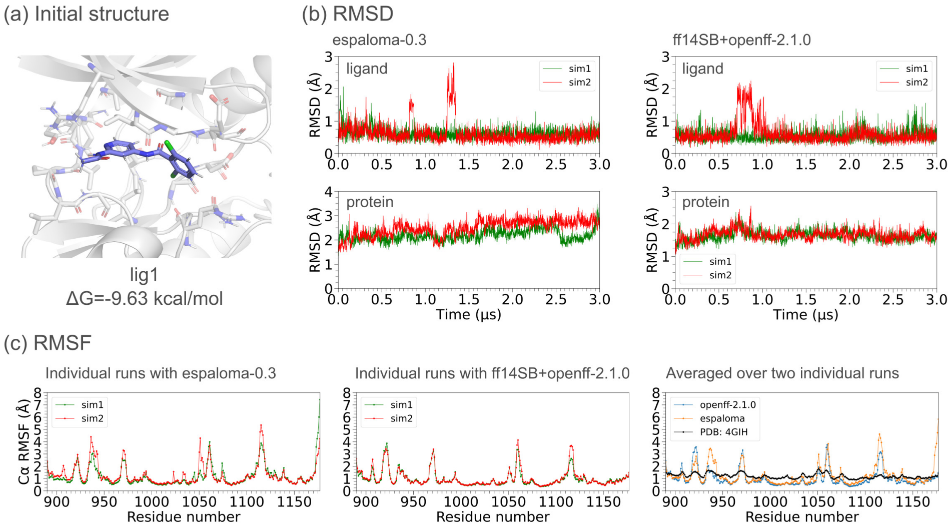

Figure S 14. espaloma-0.3 is robust and capable of stable long-time MD simulation for the Tyk2 protein-ligand complex system. Multiple 3 microsecond of MD simulations were conducted on the Tyk2 protein-ligand complex system to explore the stability of `espaloma-0.3`. (a) We show the initial structure of Tyk2 complexed with ligand #1. Two protein-ligand complex MD simulations were performed using `espaloma-0.3` to self-consistently parametrize both the protein and ligand, and `openff-2.1.0` and Amber ff14SB to parametrize the ligand and protein, respectively. (b) The root-mean square deviation (RMSD) profile of the heavy ligand atoms and protein Cα atoms are reported. The trajectories were aligned with respect to the binding pocket residues (within 4 Å from the initial ligand pose) before computing the ligand RMSD. Similarly, the protein Cα atoms excluding the first and last 5 residues, were used to align the protein trajectories before the Cα RMSD calculation, with the first and last 5 residues excluded from the RMSD computation. (c) The root-mean square fluctuation (RMSF) profile of the protein Cα atoms are reported. The experimental RMSF derived from isotropic B-factors of Tyk2 X-ray crystal structure (PDB ID: 4GIH) is shown for reference (see **SI Section H** for more details).
